# Free-form optimization of nanophotonic devices: from classical methods to deep learning

**DOI:** 10.1515/nanoph-2021-0713

**Published:** 2022-01-12

**Authors:** Juho Park, Sanmun Kim, Daniel Wontae Nam, Haejun Chung, Chan Y. Park, Min Seok Jang

**Affiliations:** School of Electrical Engineering, Korea Advanced Institute of Science and Technology, Daejeon 34141, Korea; KC Machine Learning Lab, Seoul 06181, Korea; School of Electrical Engineering, Soongsil University, Seoul 06978, Korea

**Keywords:** adjoint method, free-form optimization, machine learning, photonic device design, reinforcement learning

## Abstract

Nanophotonic devices have enabled microscopic control of light with an unprecedented spatial resolution by employing subwavelength optical elements that can strongly interact with incident waves. However, to date, most nanophotonic devices have been designed based on fixed-shape optical elements, and a large portion of their design potential has remained unexplored. It is only recently that free-form design schemes have been spotlighted in nanophotonics, offering routes to make a break from conventional design constraints and utilize the full design potential. In this review, we systematically overview the nascent yet rapidly growing field of free-form nanophotonic device design. We attempt to define the term “free-form” in the context of photonic device design, and survey different strategies for free-form optimization of nanophotonic devices spanning from classical methods, adjoint-based methods, to contemporary machine-learning-based approaches.

## 1 Introduction

Nanophotonic devices control the behavior of light on the subwavelength scale by harnessing the interaction of nanometer-scale objects with light. Thanks to the rapid development of nanofabrication techniques, various nanophotonic devices have been implemented in reality, revolutionizing many subfields of photonics such as optoelectronics [1], [2], [3], imaging [4], [5], [6], information processing [7], [8], [9], [10], [11], metamaterials [12], [13], [14], and metasurfaces [15, 16]. Modern nanophotonic devices often require not only sophisticated controls over the phase, amplitude, and polarization of light but also multiple functionalities encoded in various degrees of freedom (DoF) including wavelength [17, 18], the angle of incidence [19, 20], and other external tuning parameters [21], [22], [23], [24].

**Figure 1:**
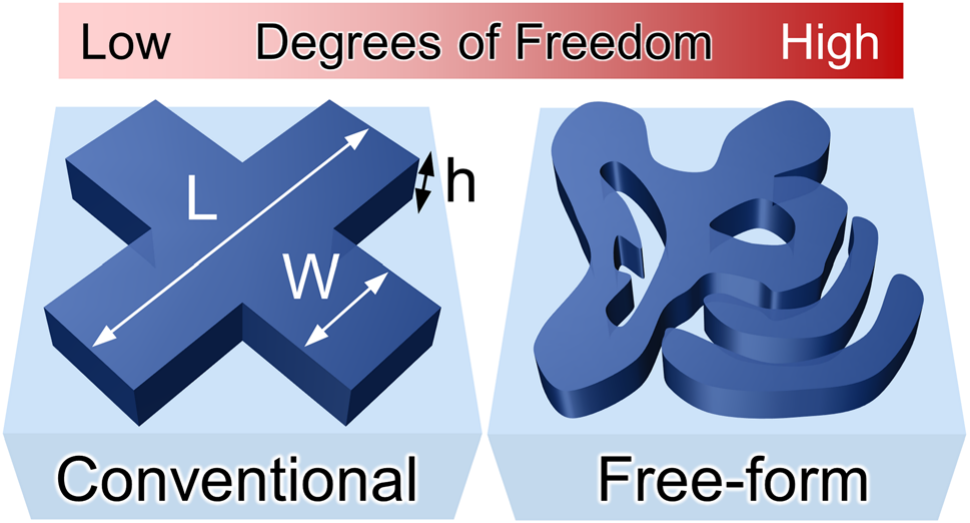
Comparison between conventional (left) and free-form (right) design schemes. In conventional design methods, photonic devices are composed of elements with fixed primitive shapes parameterized by a small number of variables. In contrast, a free-form design scheme allows for non-trivial shapes and topology modifications, exploring a much larger portion of the design space.

The growing demand for high-performance multi-functional nanophotonic devices calls for the development of an efficient device design and optimization strategy. Traditionally, nanophotonic devices are often built using physical intuitions and then fine-tuned via extensive parameter search. Specifically, conventional photonic devices are composed of elements with fixed primitive shapes such as circles or rectangles as exemplified in the left panel of Figure 1, and the optimizations of them are often performed by altering only the sizes and the positions of the shapes [25], [26], [27]. Although finding the optimum design in the constrained design space may be relatively simple, the outcome of the optimization does not guarantee a competitive performance compared to the global optimum. This has led researchers to consider more complex geometries to fully explore the photonic design space.

Thanks to both the exponential growth of computing power and the emergence of revolutionary computational methods, the field of nanophotonics has reached a point where a free-form design can be intensively studied and realized [28], [29], [30], [31], [32], [33], [34], [35], [36], [37], [38]. A free-form design strategy, as opposed to a traditional design method that is limited to fixed primitive shapes, allows the elements of a nanophotonic device to have non-trivial shapes as illustrated in the right panel of Figure 1. Furthermore, in the free-form optimization process, both the geometric shape and the topology of the structure can be freely revised, allowing access to a substantially greater design space compared to the traditional methods. But there are prerequisites to design a device via free-form optimization: an efficient but expressive parametric representation of the device design space, and an intelligent and powerful optimization algorithm to tame the immense design space enforced by the free-form design.

In this review, we thoroughly survey various free-form optimization methods that have been employed in nanophotonic device design with a focus on their recent advances. In Section 2, we discuss the definition of free-form design in the context of explicit and implicit design representations in order to answer the question “how free is free-form.” The design space of an optimization task grows exponentially with DoF, making the design problem difficult to solve. In Section 3, we introduce methods to reduce the dimensionality of a design problem. We then review classical approaches for solving photonic design problems with relatively fewer DoF in Section 4, where we explain the details of each method and introduce exemplary works that employ each algorithm. In Section 5, we discuss the theory behind the adjoint-based method and how it can be used to optimize nanophotonic devices on a large scale. Section 6 discusses machine learning-based approaches, which have revolutionized the field of nanophotonics as well as other branches of science. Although these free-form optimizations provide a great potential to nanophotonic device designs, the optimized structure could be susceptible to fabrication errors or even impossible to fabricate. Hence, in Section 7, the strategy for evaluating fabrication feasibility in free-form devices is highlighted. Finally, in Section 8, we provide an outlook for the emerging field of free-form nanophotonic device design. We also provide a grand summary of the optimization methods and the previous works on photonic device design discussed in this review in Tables 1 and 2, respectively. Table 1 outlines the key idea and pros/cons of each optimization method. Table 2 summarizes the optimization method, the device representation method, and the DoF of each design problem.

**Table 1:** The key idea and pros/cons of various optimization methods for nanophotonic device design.

Methods	Key idea	Pros	Cons
Classical (GA, PSO)	Nature-inspired population-based optimization	Easy implementation	Difficult to handle large DoF problems
Classical (conjugate gradient)	Design space search through conjugate directions	Fast convergence	Can fall into poor local optima
Adjoint-based	Lorentz reciprocity	Can handle huge DoF	Highly dependent on the initial geometry
ML (discriminative)	Finding mapping between device parameters and optical response	Can conduct diverse tasks once a network is fully learned	Large data set required for network training
ML (generative)	Learning distributions of high-performance device	Can generate multiple designs with fine performance	Many inferences required to generate an outstanding device
ML (RL)	Training of an intelligent agent by learning optimal state-action value function.	No pre-computed dataset required	Requires large computing resources
ML (physics assisted)	Minimizing Maxwell residue	Guaranteed to agree with the governing equations.	Difficult implementation

**Table 2:** The optimization method, the device representation method, and the DoF of various nanophotonic device design problems. Works without a clear statement on the design DoF, most of which are based on the level-set representation method, are not included (a: continuous – binary pushed, b: binary, c: continuous, d: discrete).

Optimization method	Device type	DoF	Representation method	Ref. #
Exhaustive search	Grating coupler	2^c^	PCA reduced geometric parameters (DoF: 5 → 2)	[55]
Classical (GA)	Broadband absorber	3^c^	Fourier spatial density function	[73]
Classical (GA)	Photonic nanojet	9^c^	Geometric and material parameters	[72]
Classical (GA)	Metasurface	225^b^	2D grid (15 × 15)	[71]
Classical (GA)	Polarization rotator	280^b^	2D grid (8 × 35)	[74]
Classical (PSO)	Fabry–Perot cavity	3^c^	Geometric parameters	[80]
Classical (PSO)	Broadband absorber	6^c^	Geometric parameters	[77]
Classical (conjugate gradient)	Multilayer nanoparticle	4^c^	Geometric and material parameters	[88]
Classical (conjugate gradient)	Multilayer nanoparticle	6^c^	Geometric parameters	[87]
Classical (GA, PSO, conjugated gradient)	Plasmonic waveguide	20^c^	Control points of Bezier curve	[79]
Classical (GA, PSO, ASA, DE)	High NA metalens	4^c^	Geometric parameters	[91]
Adjoint-based	Waveguide bend	1805^a^	Five 2D grids (19 × 19)	[129]
Adjoint-based	Diffractive optical element	2500^a^	2D grid (50 × 50)	[112]
Adjoint-based	Multifunctional metalens	5750^a^	2D grid (5 × 1150)	[134]
Adjoint-based	Photonic crystal waveguide	16,320^a^	2D grid (40 × 408)	[125]
Adjoint-based	Microstructured optical fiber	90,000^a^	2D grid (300 × 300)	[132]
Adjoint-based	Multifunctional metalens	Up to 100,000^a^	Geometric parameters	[131]
Adjoint-based	Multifunctional spectral filter	1,000,000^a^	3D grid (100 × 100 × 100)	[135]
Adjoint-based + classical (PSO)	Diffractive metagrating (tunable)	8^c^ (PSO) 400^a^ (adjoint)	Geometric parameters (PSO) 1D grid (adjoint-based)	[78]
ML (discriminative)	Broadband absorber	5^c^	Latent encoding of geometric and material parameters (DoF: 10 → 5)	[58]
ML (discriminative)	Multilayer nanoparticle	8^c^	Geometric parameters	[160]
ML (discriminative)	Metagrating	16^c^	Geometric parameters	[166]
ML (discriminative)	Waveguide splitter	400^b^	2D grid (20 × 20)	[167]
ML (discriminative) + classical (GA)	Diffractive metagrating	9^c^	Reciprocal space 2D grid (3 × 3) (DoF: 64 × 64 → 3 × 3)	[59]
ML (discriminative) + classical (GA)	Light emitting diode	10^c^	Geometric and material parameters	[161]
ML (generative)	Metasurface	4096^b^	2D grid (64 × 64)	[180]
ML (generative)	Metasurface	4096^b^	2D grid (64 × 64)	[38]
ML (generative)	Diffractive metagrating	16,384^b^	2D grid (64 × 256)	[35]
ML (RL)	Color-generating element	4^d^	Geometric parameters	[193]
ML (RL)	Broadband absorber	6^d^	Geometric and material parameters	[195]
ML (RL)	Metasurface hologram	8^d^	Geometric and material parameters	[194]
ML (RL)	Diffractive metagrating	64^b^	1D grid	[196]
ML (RL)	Multilayer spectral filter	84^d^	Geometric and material parameters	[201]
ML (physics-assisted)	Metalens	3^c^	Geometric and material parameters	[205]
ML (physics-assisted)	Diffractive metagrating	256^b^	1D grid	[128]
ML (physics-assisted)	Invisibility cloak, Wave rotator	80,000^c^	Material parameters (four parameters at 20,000 positions)	[217]

## 2 How free is free-form

Even though there have been many literatures for free-form optimization of photonic devices including metalenses [18, 39, 40], beam deflectors [41, 42], power splitters [43, 44], and wavelength demultiplexers [45], it was often unclear what kind of photonic structures are to be considered as the results of free-form design, because there is no canonical definition of “free-form.” The definition of free-form design is inevitably entangled with device design representations, as they determine the accessible design space. In this section, we introduce explicit and implicit design representations and suggest the meaning of free-form design in each context.

**Figure 2:**
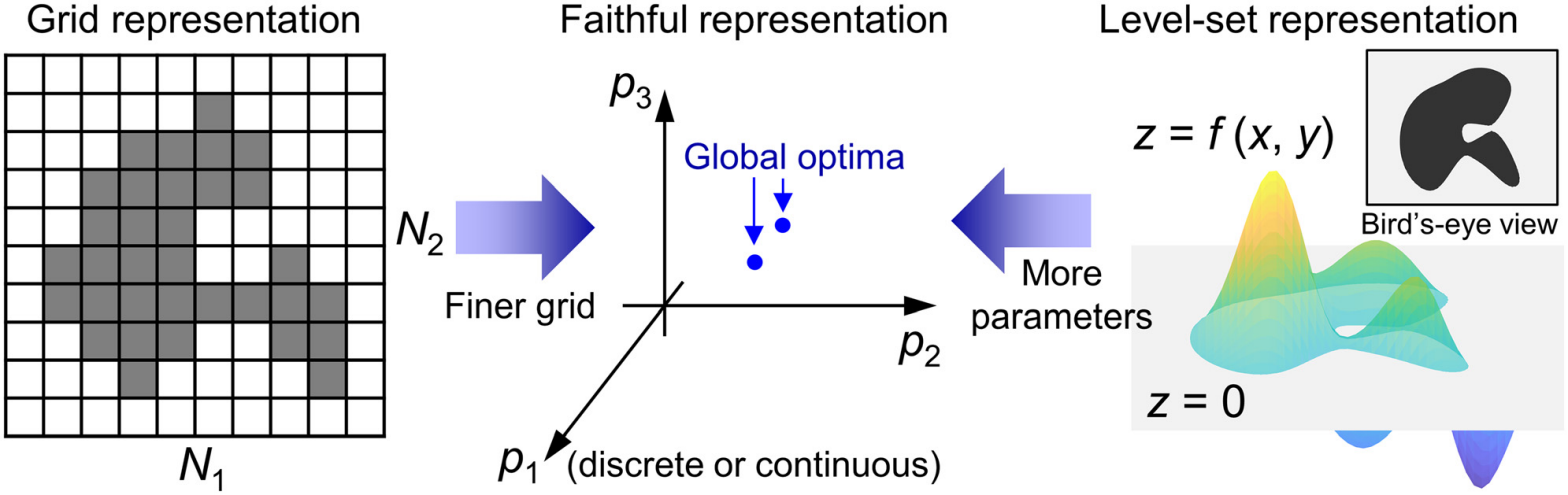
Examples of various representations for 2D beam deflectors. A grid representation (left) is an example of an explicit representation of a device, which becomes closer to a faithful representation as the grid becomes finer. A level-set representation (right) is an example of an implicit representation, whose expressiveness is determined by the number of basis functions used and therefore approaches a faithful representation as the basis covers more and more functional space. In a faithful representation (middle), each point in the representation is bijectively mapped to a device, therefore the representation space coincides with the whole device design space and there can be one or more global optima in the space.

Ideally, we want to have a “faithful” representation of the whole design space of a given family of photonic structures, say a 2D beam deflector, as shown in the middle panel of Figure 2, where for the sake of simplicity we only denoted a couple of global optima for a given set of design parameters (*p*_1_, *p*_2_, *p*_3_). This means that in the representation space we have all the structures we want to explore and only such structures. Furthermore, we want to have a disentangled parametrization of such a representation space, i.e. a cartesian coordinate system with each axis corresponding to a meaningful design parameter. Dimensionality reduction of the raw design space can be regarded as an effort to obtain such a faithful representation space, the detail of which is covered in Section 3. But as of now, even with additional optimization objectives included for the regularization for disentanglement, we cannot yet obtain neither such a representation space nor such a coordinate system, which is still an active area of research.

To reach the “promised land” of a faithful representation, one starting point is an explicit grid representation of a design space. For example, consider a 2D beam deflector in an explicit grid representation shown in the left panel of Figure 2, where the design space is divided into *N* = *N*_1_ × *N*_2_ rectangular grids. Each grid node can have a value of either 0 (void) or 1 (material), which makes the optimization problem into a binary combinatorial problem. In this scheme, if *N* is sufficiently large so that each grid node can represent subtle behaviors of electromagnetic responses, the representation can exhibit all possible structures in the design space. Note that *N* is dependent on each specific design optimization problem, and typically limited by the extent of simulation accuracy: For example, in FDTD (finite-difference time-domain) method, the rule of thumb for the grid size is to set it as *λ*/20. An upside of this representation is that, in theory, with an infinitely fine grid we can represent any photonic structure; but of course, this is unrealistic and the increase of dimensionality as we make a grid finer runs into a practical problem. What makes these design schemes “free” is that it is straightforward how such a scheme represents a device, and one only needs to focus on how to devise an algorithm that can obtain an optimal structure by exploring the design space. In that sense this is a free-form design, i.e. it frees a researcher from thinking about how to represent a device and instead let the researcher work on the algorithmic side of the optimization, which can be universally applied to various device structure optimizations, hopefully.

On the other hand, there are implicit design representation methods, such as level-set basis functions [46, 47], spline curves [48, 49], or Fourier spectral basis [50, 51]. These methods try to capture the “axes” of the true design space via domain expertise. The right panel of Figure 2 illustrates a level-set representation of a 2D beam deflector (see Section 7 for more about the level-set method). These methods have complementary ups and downs compared to grid representations: we have a controllable number of parameters, but often such a representation is not enough to capture the full design space, and as we increase the number of parameters, we face the same problem as a finer grid representation: not enough computational power to deal with a large number of parameters. It would be worthwhile to note that any implicit design representation can be projected into an explicit grid representation by using geometry mapping [52], where boundary features smaller than the size of a grid are either deleted or filled, resulting in a staircase boundary; Therefore, the possible design space of an implicit design representation is a subset of full design space constructed by the binary combinatorial representation. An implicit design method can also be considered as “free” because it provides us with structures that are largely free from primitive geometric shapes such as straight lines and polygons.

So, we say that there are two meanings of “free” when we say a free-form design: free from hand-engineered design schemes, or free-from geometric shapes in the real space. The two schemes approach a faithful representation of the whole design space as their number of parameters increases, i.e. the number of grids in the grid representation and the number of spectral basis vectors in the Fourier representation. But in practice, we cannot increase the number indefinitely and there should be a tradeoff between the design space coverage by increasing the parameter numbers. Therefore, a smart way to discover the right subspace of the design space either by devising a good algorithm in the case of the grid representation, or by providing an expressive parametric representation in the case of an implicit method, needs to be established.

In general, finer grid spacing in the grid representation is correlated with a higher potential figure-of-merit (FoM) of the optimized structure, but there may be a practical upper bound to “how fine it should be” in order to achieve a FoM that is close to the global optimum. If a grid spacing of *λ*/20 attains the global optimum but *λ*/15 does not, we can claim that the grid spacing finer than *λ*/20 is the upper bound of the problem. Because the induced polarization ( $P_{\text{ind}}=\alpha E$ ) due to a small feature (e.g., grid spacing) is dependent on the shape-dependent polarizability (*α*) and the electric field (**E**), this bound may be affected by the material’s dielectric constant and the optimization objective. Greater dielectric constants, including those found in metallic materials, may require significantly finer grid spacing. Also, a high-quality factor design may also increase induced polarization due to the greater electric field. In this regard, defining a single magic number for suitable grid spacing in free-form designs that cover all the cases is next to impossible. However, in practice, where the material is dielectric and the quality factor is less than 10, the *λ*/20 grid spacing seems to be enough for discovering a high-performance free-form structure [39] with the optimization methods that we will discuss in this paper. Non-staircase-type methods such as the finite element method (FEM) may have a lower bound compared to the rectangular, staircase-like grid.

Many photonic design researches have benefited from free-form optimization due to its ability to freely adjust boundary and connectivity of geometric shapes. However, even with its superior performance potential, not all design problems require free-form optimization. For example, fabrication constraints can be implied in the design steps, and it is relatively difficult to integrate a free-form optimization scheme with electromagnetic formulations. Moreover, an optimized design with less-complicated representations can be used as an initial point of free-form optimization. For these reasons, we showcase these weak free-form designs in Section 4, with derivative-free algorithms that can be applied to optimization problems having a small number of design parameters.

## 3 Dimensionality reduction

The size of the design space does not always match that of the true optimization space. As the size of the design space increases exponentially with the number of parameters to tune, reducing the number of dimensions of the design space can provide a big advantage. For population-based methods, the design space size is directly related to the computation time for obtaining the optimal solutions, and in the field of machine learning the curse of dimensionality is widely recognized [53]. In this section, we discuss three representative methods commonly used in nanophotonic device optimizations to reduce the number of dimensions of the design space.

### 3.1 Principal component analysis

**Figure 3:**
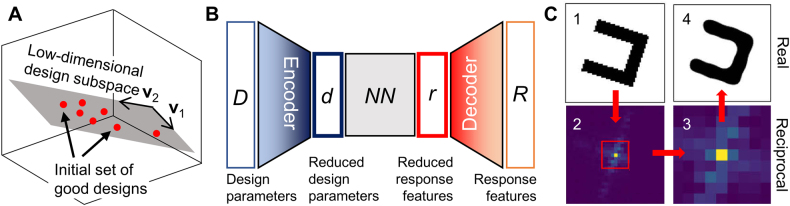
(**A**) A schematic of employing PCA in a three-dimensional design space. PCA reveals the plane where the good designs reside, thus reducing the dimensionality. Figure adapted from Melati et al. [55]. Licensed under CC BY 4.0. (**B**) Dimensionality reduction with autoencoder. Design parameters and optical response features are encoded into reduced parameters. (**C**) Encoding a two-dimensional geometry into a low dimensional Fourier space. (1: Original image, 2: Original image in the reciprocal space, 3: Low dimensional image obtained by removing high-frequency components. 4: Image recovered from inverse Fourier transforming image in 3). **C** Figures are adapted with permission from [59]. Copyright 2020, Optical Society of America.

First suggested by Karl Pearson in 1901 [54], principal component analysis (PCA) is a classical approach to dimensionality reduction, widely used even before the invention of computers. Unlike the dimensionality reduction using physical intuitions, PCA comes at the expense of accuracy; it aims to obtain the principal components affecting the features through the calculation of covariance among the normalized parameters. Each principal component is formed by taking the linear combination of the original parameters, and the set of principal components becomes the orthogonal basis spanning the subspace of interest. Unless the number of principal components is equal to the number of original parameters, information loss is inevitable in PCA but the selection of original parameters with highest impact on the feature minimizes the loss. The work by Melati et al. [55] provides a prominent example of utilizing PCA in nanophotonic device design as illustrated in Figure 3A. They designed a grating coupler structure defined with five structure parameters. The authors first obtained a sparse collection of good designs, then PCA was operated on these designs to find the parameters that lead to a good design. The results from PCA showed that good designs reside on a subspace spanned by two orthogonal basis vectors. Just from an exhaustive exploration in the reduced two-dimensional subspace, the authors found optimal designs that have a coupling efficiency competent to that of the previously reported design.

### 3.2 Autoencoder

PCA finds a subspace where the good designs reside. However, a prior dataset of good designs is required to operate PCA on the problem space, and the information of designs with low FoM is neglected. Also, one critical problem of PCA is that the basis spanning the subspace is restricted to the linear combination of the original design parameter. In order to overcome the aforementioned issues, a neural network-based approach using autoencoder was introduced [56, 57]. An autoencoder is a neural network that aims to extract the information while removing the noise from the data. A classical autoencoder receives an input and tries to reconstruct the input value at the output end of the network, while having a bottleneck layer in between the input and output end. The bottleneck layer has a smaller number of dimensions than the input/output size, so the input data (original representation) is encoded/compressed to the bottleneck layer (reduced representation) through a neural network, often called an encoder. The output is decoded from the reduced representation through a neural network, often called a decoder. For its utilization in nanophotonics devices, we focus on the fact that both the design parameters and the optical response can be described by a representation of a lower-dimensional space. For example, if we are dealing with the transmission through a multilayer film where the data type is a transmission spectrum sampled with a step size of 1 nm between 500 and 700 nm, the number of dimensions of the faithful representation space is much smaller than 201 since the transmittance at 500 nm is likely to be similar to the transmittance at 501 nm than with 700 nm. In case of design parameters, multiple structures may exhibit the same optical response due to translational symmetry or non-trivial physical reasons which implies the existence of a reduced representation of the design space. The work by Kiarashinejad et al. [58] provides a good example of utilizing autoencoder to nanophotonic device designs. They trained a neural network that maps vectors in design space/optical response to a reduced design space and an optical response space respectively as illustrated in Figure 3B. A more detailed mapping was established between the data points in the reduced design space, the reduced response space, and the original response space. Through optimization within the reduced design space, the authors could reduce the computation load.

### 3.3 Dimensionality reduction in reciprocal space

An alternative approach utilizing the Fourier space can be made to efficiently represent a 2D binary image as demonstrated by Liu et al. [59]. They accomplished the dimensionality reduction by mapping the design space to the reciprocal space using Fourier transformation of its level function. When a 2D image with dimension of *N*_3_ × *N*_3_ is mapped to the reciprocal space, a complex *N*_3_ × *N*_3_ matrix is obtained without a reduction in dimension. However, the general topology of the design is encoded near the origin of the reciprocal space whereas the numbers in the higher-order term accounts for the small features. Hence, the authors kept only the 3 × 3 region centered at the origin from the 64 × 64 matrix in reciprocal space to reconstruct the binary image as illustrated in Figure 3C. Each component in the 3 × 3 matrix is a complex number, but in order for the level set function to be real-valued, an additional constraint was levied, cutting the DoF by another half, resulting in nine DoF to define a 64 × 64 binary image. Using the topological encoding scheme, the authors were able to tackle the inverse design and the optimization of non-paraxial diffractive optical elements using the fully connected network and population-based algorithms. In their paper, a further reduction of DoF by the inclusion of additional symmetries and the continuity in latent reciprocal variables were also discussed.

## 4 Classical methods

In the early era of device design, researchers used to create designs based on physical intuitions combined with simple parameter sweeps. With the increase of computational power and advances in simulation methods in the past several decades, researchers in many scientific and engineering disciplines were able to perform numerical optimizations for structures having complex geometry that handle a previously inaccessible and relatively large design space. As a representative example, in the early 1990s, spacecraft antennas were designed using evolutionary optimization algorithms [60], [61], [62]. We would like to call the optimization methods used in the early history of optimizations as “classical methods” to differentiate them from more recent approaches such as the adjoint-based methods and machine learning algorithms which will be discussed in later sections.

**Figure 4:**
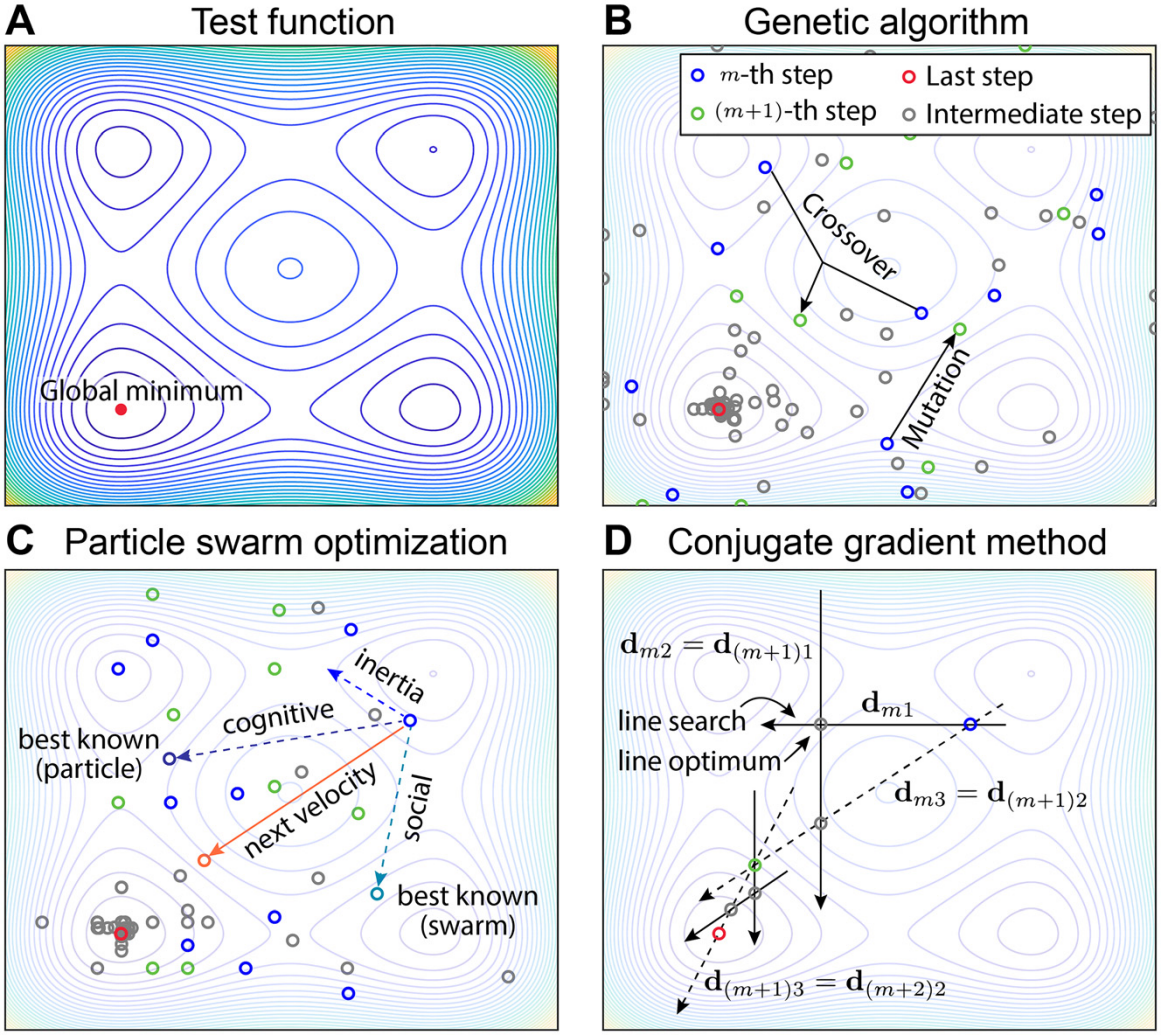
Illustration of classical optimization methods and a test function (Styblinski–Tang) to which the methods are applied. (**A**) Contour plot of test function. Global minimum is indicated as a red dot. (**B**) Schematic of GA applied to the test function. The black arrow merged between two blue circles represents crossover operation. The black arrow from the single blue circle represents mutation operation. For (**B**–**D**), evaluation points during optimization at current (*m-*th), next ((*m* + 1)-th) and last step are indicated as blue, green, and red circles, respectively. Other intermediate evaluation points are indicated as gray circles. (**C**) Schematic of PSO applied to the test function. Historical best-known particle from a swarm and its own are shown in turquoise and purple circles. The velocity vector representing inertia, cognitive, and social terms are indicated as blue, purple, and turquoise dotted arrows, respectively. (**D**) Schematic of conjugate direction method applied to the test function. The solid and dotted black arrows indicate the direction of line searches at each corresponding step. The dotted black arrows represent the newly added search direction at each corresponding step. Note that some of the evaluation points in (**B**–**D**) were modified from actual locations, for an illustration purpose.

For contemporary nanophotonic device design, these classical optimization methods are still important, because they can be used as an initial approach for solving simplified versions of complex geometry optimization problems, owing to its easy implementation. The optimized design in a simplified parameterization would be used as an initial point for optimization with more complex parameterizations with higher DoF. Moreover, for a moderate number of design parameters (lower than a few tens), classical methods tend to show superior performance than the newer algorithms [63]. In this section, we review three representative classical optimization methods frequently employed in nanophotonic device design: genetic algorithm (GA), particle swarm optimization (PSO), and conjugate gradient method. GA and PSO are stochastic, global optimization, while the conjugate gradient method belongs to a family of deterministic, local optimization methods. All three methods do not require an evaluation of FoM gradients in the parameter space and are thus suitable for solving problems having highly nonlinear or non-differentiable FoMs. The overall schematics of GA, PSO, and the conjugate gradient method, which are applied to Styblinski-Tang test function (Figure 4A), are drawn in Figure 4B, C, and D, respectively. The details of each method will be discussed in the following subsections.

### 4.1 Genetic algorithm

First introduced by Holland [64] in the early 1970s, GA has been used for many optimization problems in numerous fields, including robotics [65], computer vision [66], structural mechanics [67], and chemistry [68]. Inspired by natural evolution, GA achieves optimal solutions by iterative evolution of chromosomes, each of which is a set of design parameters (each parameter is called a gene). The three primary operations of GA are selection, crossover, and mutation. A selection operation picks several chromosomes that show good performances in a pool, commonly by Roulette Wheel method [69]. A crossover operation occurs between two chromosomes determined in a selection operation. In this step, the gene sequences of two chromosomes are interchanged or combined to yield a new child chromosome, as shown in Figure 4B. The interchange and combination of superior chromosomes are expected to produce a child chromosome with a better performance. A mutation operation randomly changes the values of the genes in chromosomes after the crossover operation, as also shown in Figure 4B. Random changes of design parameters give room for escaping local extrema [70].

Thanks to its versatility, a vast range of nanophotonic devices have been optimized through GA. Those devices include plasmonic metasurfaces [71], photonic nanojets [72], broadband absorbers [73], and on-chip polarization rotators [74]. Huang et al. [72] integrated Mie theory and GA to optimize a photonic nanojet device based on multilayer microcylinders. The device consists of five-layer core–shell cylinders, having the thicknesses and the refractive indices of each layer as design parameters. The optimized microcylinder device shows ultra-long photonic nanojet with a length of 107 times its operating wavelength. Lee et al. suggested a way to combine quasi-random design representation and GA to design broadband absorbers [73]. By examining the α-Si quasi-random patterns on α-Si substrate, fabricated by wrinkle-lithography process, the authors were able to analytically derive Fourier spectral density function of quasi-random patterns, which is determined by only three parameters: the wrinkle wavelength, the material filling ratio, and the feature depth. As a result of the optimization for a broadband absorber, the fabricated device showed a 163% enhancement in the average absorption, compared to an unpatterned α-Si substrate.

**Figure 5:**
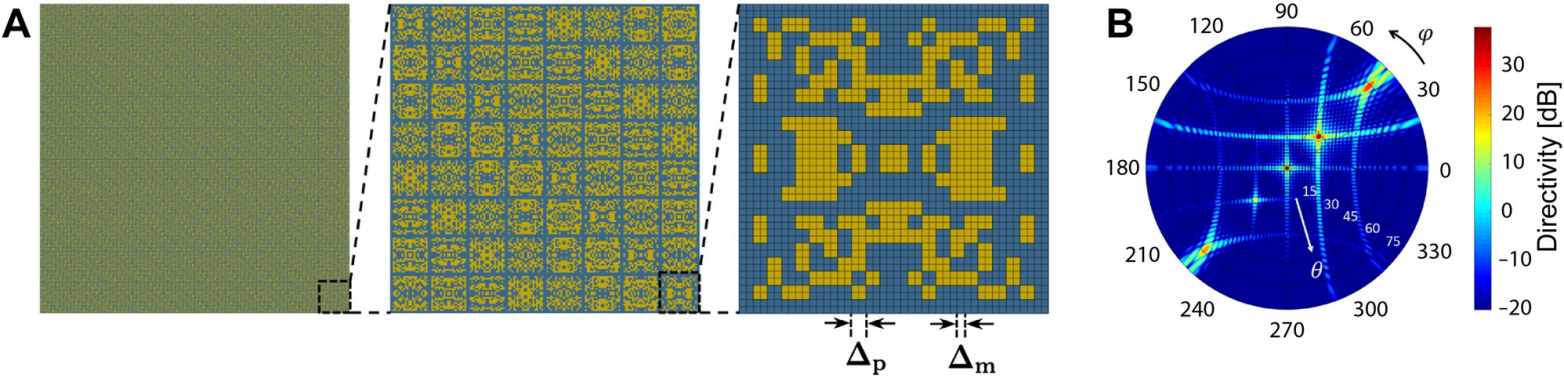
Free-form optimization of plasmonic reflective beam deflector. (**A**) Material distribution image of the proposed metasurface, which is composed of 10 × 10 periodic identical super-cells, designed to reflect a normally incident beam to the target direction. (Left) Magnified view of 8 × 8 super-cells of the metasurface (middle) and the lower-right unit-cell of the super-cell (right). (**B**) Polar reflection pattern of the designed metasurface shown in a form of directivity. **A**, **B** adapted with permission from [71]. Licensed under CC BY 4.0.

Jafar-Zanjani et al. [71] demonstrated the inverse design of a free-form plasmonic metasurface beam-deflector and wavelength-selective absorbers, using a significantly greater number of design parameters compared to the works mentioned above. In this research, the authors proposed an adaptive genetic algorithm (AGA), a slightly modified version of GA, to solve the multi-objective optimization problems. For multi-objective design problems, the conventional GA sets weights for each objective and sums those to yield a single scalar FoM. Instead of optimizing this integrated objective at once, AGA first set only high priority objectives as FoM. Then, the initial population for AGA is generated using the conventional GA. After that, according to stop criteria, FoM is updated to include subsequent objectives with less priorities. Iterating these steps allows AGA to converge to near-optimal solutions with satisfying both high-priority objectives and the ones with low priority. For plasmonic reflective beam-deflectors, the metasurface consists of 10 × 10 periodic identical supercells (the left panel of Figure 5A), with each supercell consisting of 8 × 8 unit cells (the middle panel of Figure 5A). The unit-cell is then divided into 20 × 20 = 400 square patches as shown in the right panel of Figure 5A, and each patch is binary-coded to yield design parameters. Using AGA, total eight distinct unit-cells are optimized to cover 2*π* phase range required to shape the phase profile generating a deflected beam toward desired target direction (*θ*_0_, *φ*_0_) = (30°, 45°). The designed metasurface shows the main lobe at (*θ*, *φ*) = (28.6°, 45°), which is close to the target direction, as shown in Figure 5B. Similar parameterization was used in their wavelength-selective metasurface absorber design, which divides the unit-cell into 15 × 15 = 225 square binary patches. The optimized metasurface shows more than 80% absorption for 10–19 μm wavelength range, and more than 0.7 reflection amplitude for 400–700 nm.

### 4.2 Particle swarm optimization

Inspired by the social behavior of bird swarms, PSO was introduced by Kennedy and Eberhart in the 1990s [75]. In the PSO scheme, each individual in the population referred to as a particle has position and velocity values. At each iteration of PSO, the position and velocity values are updated through the governing update equations [76]. A standard form of the velocity update equation consists of an inertia term, a cognitive term, and a social term, as schematically illustrated in Figure 4C: The inertia term represents how much of the previous velocity is transferred to the next velocity value, indicated as a blue dotted arrow in Figure 4C. The cognitive term steers the particle toward the personal best, which is the best point the particle has found in its record of the search paths, indicated as a purple dotted arrow in Figure 4C. Like the cognitive term, the social term adjusts the direction of the particle velocity toward the swarm best, which is the best point the overall swarm has found in their record of the search paths, indicated as a turquoise dotted arrow in Figure 4C. The next velocity (solid orange arrow) is formulated as a weighted sum of the inertia, cognitive, and social term. After the velocity is updated, the position is updated using the previous position and updated velocity values.

**Figure 6:**
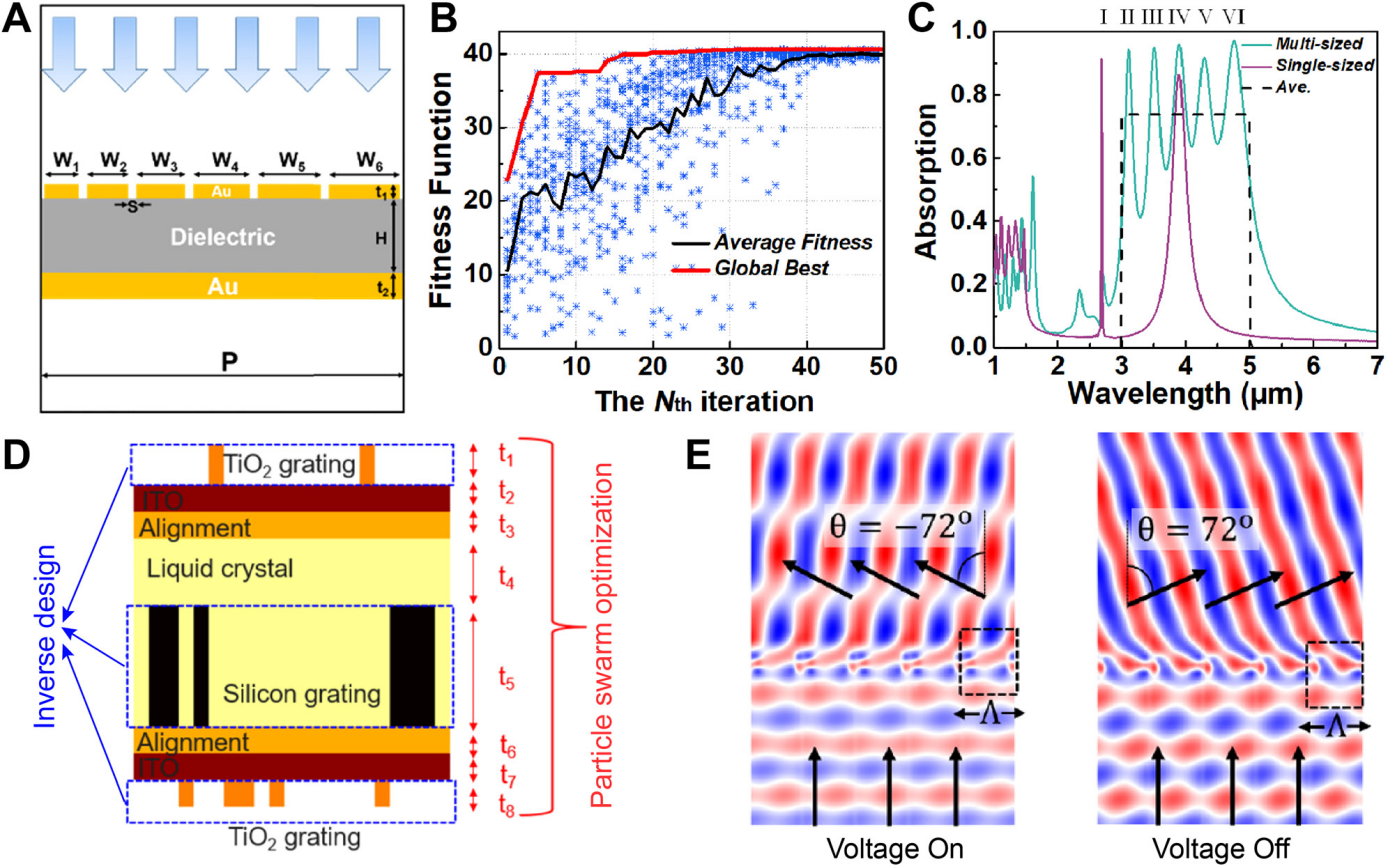
Nanophotonic device optimization using PSO and its results. (**A**) Schematic of three-layer PMA structure. (**B**) Fitness function evolution through PSO progress. The black line indicates average fitness value at each iteration. The red line indicates the largest fitness value observed since the first iteration. The blue stars represent the fitness values of all particles at each iteration. (**C**) TM-wave absorption spectra for the multi-sized nanostrips (green line) and the single-sized nanostrip absorber (purple line). Mark I to VI corresponds to the LSPR peaks induced by individual nanostrip in multi-sized nanostrip absorber. The average absorption of 73.7% for the multi-sized nanostrip absorber is indicated as black dashed line. **A–C** Figures are adapted with permission from [77]. Copyright 2019, Optical Society of America. (**D**) Schematic of liquid crystal-based electrically tunable beam switching metasurface. (**E**) Real parts of the electric fields plot for the voltage-on (left) and voltage-off (right) states. **D, E** adapted with permission from [78]. Copyright 2020, American Chemical Society.

PSO has been widely adopted in designing a variety of nanophotonic devices spanning broadband absorbers [77], metagrating beam deflectors [78], plasmonic light trapping structures [79], to Fabry–Perot cavities based on optical fibers [80]. Li et al. conceived multifunctional plasmonic metamaterial absorber (PMA) for infrared imaging [77], which has an advantage of significantly negligible crosstalk compared to the conventional micropolarizer approach. The proposed PMAs work as polarization-dependent absorbers for broadband wavelength. The absorber structure comprises three layers, each consisting of several Au nanostrips, a dielectric spacer, and an Au back reflector, respectively, as shown in Figure 6A. The width of the Au nanostrips is set to form an arithmetic sequence, and a constant space is placed between neighborhoods. The PSO optimized dielectric layer thickness, space between nano strip neighborhoods, and widths of the nanostrips. The FoM of optimization was set to the ratio of the sum of absorption by transverse-magnetic (TM) polarized light over the one by transverse electric (TE) polarized light, in the 3–5 μm wavelength range. Initially, the global best fitness value was smaller than 25, but at the end of the optimization, the fitness value was over 40, as shown in Figure 6B. It was observed that all particles have converged to an optimum point, exhibiting almost the same fitness values at the end of the optimization. The absorption spectra of the optimized multi-sized (green line) and single-sized (purple line) nanostrip structures under a normally incident TM wave are shown in Figure 6C. The absorption peaks marked as I-VI are the results of localized surface plasmon resonances, each of which is induced by an individual nanostrip. While the single-sized nanostrip structure only shows 18.5% average absorption, the optimized multi-sized nanostrip structure shows 73.7% average absorption (black dashed line in Figure 6C).

The work by Chung et al. [78] for optimization of liquid crystal-based electrically tunable beam switching metasurface utilizes a hybrid method integrating PSO and the adjoint-based method. The device structure consists of triple gratings (TiO_2_/Si/TiO_2_), ITO, alignment, and liquid crystal layers, as shown in Figure 6D. The active tuning mechanism based on liquid crystal lies in the change of the refractive index tensors depending on the voltage state. When the voltage is on, the liquid crystal director is perpendicular to TE mode electric fields, and when it is off, the liquid crystal director is parallel to the electric fields. These alignments maximize the effective refractive-index change that the incident light experiences while traveling. The patterns for triple gratings were inverse-designed using adjoint sensitivity analysis and the thicknesses of triple gratings, ITO, alignment, and liquid crystal layers were optimized by PSO. PSO governed overall optimization progress. The particles in the swarm were initialized with different layer thickness parameters. Then, within each particle, inverse-design optimization was conducted to design grating patterns. The authors reported that the global optimization by PSO was crucial in their optimization, since in their single-grating optimization, the optimized structure showed 53% switching efficiencies, but without PSO, it only showed an efficiency of 37% for 15° steering angle. A triple-grating optimization results in a device exhibiting ultrawide angle deflection of 144° with diffraction efficiencies of 62 and 76% for voltage on and off states. The electric field distributions in Figure 6E show clear diffracted waves along each desired direction.

### 4.3 Conjugate gradient method

GA and PSO stated in the previous subsections are classified as evolutionary algorithms, invented from a motivation inspired by the behavior or movements of entities in nature. On the other hand, the conjugate gradient method stems from optimization methods initially analyzed for quadratic problems. The idea behind the conjugate gradient method is to find a solution following along a set of conjugate directions.

Among many variations of conjugate gradient methods, here we introduce the one developed by Powell [81], which is broadly used for many disciplines [82], [83], [84]. In Powell’s method, the consecutive line searches create a new conjugate direction per iteration. An illustration of Powell’s method for two-dimensional parameter space is shown in Figure 4D: The dimension of the parameter space is equal to the number of conjugate directions. For the *m*th step of the conjugate gradient method, a line search is done through two conjugate directions **d**_*m*1_ and **d**_*m*2_ (for *m* = 1, **d**_*m*1_ and **d**_*m*2_ are set as two normal coordinate directions). After the line searches are done, the optimal point is found (green circle), and the starting point of the *m*th step (blue circle) and the optimal point are connected to form a new direction **d**_*m*3_. Now, the first conjugate direction in the previous step is abandoned from the conjugate direction set and the newly created direction vector **d**_*m*3_ is added to the direction set, resulting in a set of **d**_(*m*+1)1_ = **d**_*m*2_ and **d**_(*m*+1)2_ = **d**_*m*3_. This procedure is repeated until the local optimum (red circle) is found.

Like GA and PSO, the conjugate gradient method is also a derivative-free algorithm, meaning that it does not require an analytical differentiation of the objective function throughout optimization processes. The conjugate gradient method guarantees convergence to the global optimum within finite steps for quadratic problems, therefore having “quadratic termination” [85]. For general, non-quadratic problems, the conjugate gradient method may fall into local optimum, and the quality of the solution highly depends on the initial points. However, this issue may be overcome by conducting multiple optimizations starting from random (cold-start) or previously found (warm-start) initial points, which increases the reliability of the optimization methods and allows escaping from the local optimum [86].

The conjugate gradient methods have been utilized in nanophotonic device optimizations with relatively small design spaces which can be typically parametrized with less than 10 design variables. Qui et al. [87] optimized nanospheres that consist of alternating layers of Ag and SiO_2_. In their work, the FoMs were set as the average scattering, absorption, and total cross-section in the target frequency range of 400–600 and 600–800 nm, resulting in six separate optimizations. Interestingly, all optimizations resulted in nanospheres with only two layers-Ag coated SiO_2_ bilayer-even though the spheres were allowed to have up to six layers of materials. Ye et al. [88] also tackled the problem of nanoparticle scattering using the conjugate gradient method. In their work, the authors optimized geometric parameters (the core diameter and the shell thickness) and a material parameter (gain index) of SiO_2_ shell surrounding a metal core to obtain selective RGB scattering. The FoM was set as a function of the scattering and absorption cross-sections to maximize scattering cross-section at resonant wavelength, minimize scattering cross-sections elsewhere, and minimize overall absorption cross-section. As a result, the authors recommended SiO_2_/Au and SiO_2_/Ag material combinations for a sharp selective scattering of red and green–blue, respectively.

**Figure 7:**
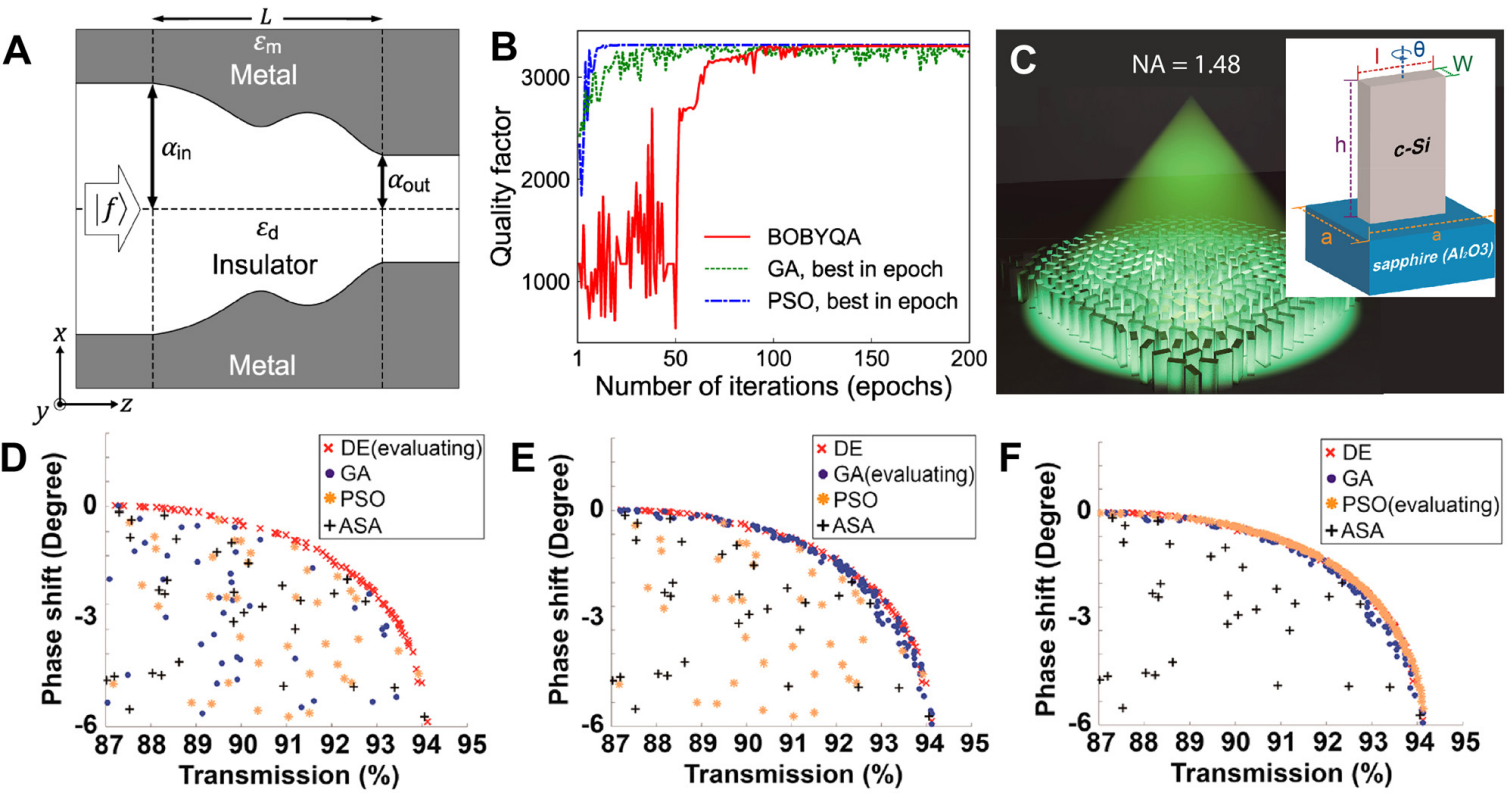
Works for nanophotonic device design depicting comparison between classical methods and a hybridized method consisting of them. (**A**) Schematic of free-form MIM light trapping waveguide. (**B**) Quality factor evolution along the iterations (epochs) of optimization. Solid red line, dotted green line, and dash-dotted blue line shows optimization progress for BOBYQA, GA, and PSO, respectively. **A**, **B** adapted with permission from [79]. Copyright 2019, American Physical Society. (**C**) Rendered image of proposed ultrahigh-NA metalens. Inset shows the unit cell composing the metalens, which is being optimized. (**D**–**F**) The population distribution showing phase shift and transmission, during optimization iterations at which DE (**D**), GA (**E**), and PSO (**F**) are evaluated. **C**–**F** Adapted with permission from [91]. Copyright 2018, American Chemical Society.

Unconstrained by pre-fixed primitive shapes, Park et al. [79] demonstrated a free-form optimization of the shape of a metal-insulator-metal (MIM) waveguide to maximize light trapping time. The concept of the MIM waveguide used here is based on “rainbow trapping” [89], which shows a concentration of light with different wavelengths at corresponding positions and thus used for spectrum splitting [90]. The waveguide shape is parameterized by the control points of Bezier curve, showing free-form structure while both endpoints are fixed as illustrated in Figure 7A. By using bound optimization by quadratic approximation (BOBYQA), which is a variant of the conjugate gradient method, Park et al. were able to identify the maximum achievable quality factor for a MIM waveguide with given length and input and output widths. Compared to conventional linearly tapered waveguides, the quality factor of the optimized structure saturates at much shorter length, and this critical length scales logarithmically with the inverse material loss.

### 4.4 Comparison of methods and their hybridization

From Section 4.1 to 4.3, we have discussed three classical optimization methods: GA, PSO, and conjugate gradient method. Since the formulation and mechanism of those methods are quite different from each other, there is no guarantee that the solutions of an optimization problem obtained from those will coincide. Conversely, if one could reach the solutions exhibiting similar FoMs using different optimization algorithms, it might be highly likely that the solution is a global optimum. The study by Park et al. [79] on MIM light trapping structures (Figure 7A) illustrates this point. Figure 7B shows the evolution of the FoM, which is the quality factor of the waveguide, as a function of optimization epochs (iterations), for GA (dotted green line), PSO (dash-dotted blue line), and BOBYQA (solid red line). Considering that the number of evaluations in single optimization epoch for GA and PSO scales linearly with the number of individuals in the population, the total evaluation number seems to be very large compared to the case of conjugate gradient method. However, the actual number of evaluations is comparable because the conjugate gradient optimization should be conducted several times with different initial points to avoid falling into local optima. The three optimization methods reached almost the same FoM, strongly supporting that the point was the global optimum. Schneider et al. also compared a variety of classical optimization algorithms in Ref. [63].

A smart combination of different optimization algorithms could lead to a better optimization performance than that of a single algorithm. Liang et al. [91] suggested a hybrid optimization algorithm (HOA) to design the unit-cell (inset of Figure 7C) of a metalens with a high numerical-aperture of 1.48 at visible wavelengths as illustrated in Figure 7C. The goal of the HOA is to push Pareto Frontier [92] of the multi-objectives, which are the transmission phase and amplitude response of the unit cell. The HOA composes of successive optimizations using several global optimization algorithms. The concept of HOA is to provide locally optimized candidates in the previous optimizer to the next optimizer as “good” initial points. Detailed procedures of HOA are as follows: The HOA first initializes the population with randomly chosen structure parameters. The population is optimized by the first optimizer differential evolution (DE) (Figure 7D). Next, a fraction of the best optimum solutions are migrated to the next optimizer. These “immigrants” work as a prior knowledge to the next population, which increases the possibility of finding the global optimum. This process is repeated for GA (Figure 7E), PSO (Figure 7F), and adaptive simulated annealing (ASA). This consecutive application of DE, GA, PSO, and ASA defines one generation. At the end of each generation, the HOA checks if the best optimum points in the four optimizers coincide, which means the optimums are close to the global optimum. If not, the HOA continues until all optimizers point to global optimum solutions. By using HOA, the width, the height, and the thickness of a c-Si nanopost and the unit-cell period were optimized. The obtained unit-cells cover 2*π* phase range with a high transmittance above 87% as shown in Figure 7F and were placed on the metalens to satisfy the phase profile required for beam focusing. As a result, the authors could design a metalens with NA = 0.98 with focusing efficiency of 67% at 532 nm wavelength. With the incorporation of immersion oil, focusing with NA = 1.48 was achievable.

As seen in the above examples, the researchers who used classical methods in their papers tend to choose a small number of design parameters for optimization. This might be attributed to the curse of dimensionality, which states that the convergence of optimization slows down dramatically as the number of design dimensions increases, even if the number of design parameters is not limited by its algorithmic characteristics. The effect of the curse of dimensionality is documented for PSO and DE in Ref. [93].

GA, PSO, and conjugate gradient methods are easily accessible in several programming language libraries. For MATLAB, GA and PSO are included in Global Optimization Toolbox, and the conjugate gradient method is included as a built-in function. For Python, numerous evolutionary algorithms, including GA and PSO, are implemented in DEAP (Distributed Evolutionary Algorithms in Python) library [94]. Among many conjugate gradient methods, BOBYQA is available in Py-BOBYQA library [95].

## 5 Adjoint-based method

The classical optimization methods covered in the previous section succeed at yielding satisfactory results for design problems with relatively small DoF. However, when the DoF increases as design problems become complicated, the derivative-free methods often suffer from tremendous computation cost, due to the curse of dimensionality. In this situation, FoM gradient-based optimization methods could be a better option. Applying the gradient-based methods for high DoF design problems requires an efficient calculation of FoM gradients. Conventionally, the finite-difference method was used to calculate FoM gradients. The finite-difference method approximates gradient as a slope of the objective function in small increments of design parameters. Since the finite-difference method calculates FoM gradients for design parameters one by one, the net calculation time scales with the number of design parameters, which hinders the optimization of devices with high DoF. To overcome this problem, an adjoint method was proposed.

The adjoint method is a numerical way of calculating FoM gradients for optimization problems in an efficient manner. This process has its roots in control theory dating back to the 1960s [96, 97], after which it was utilized in circuit theory [98] during the same era and then in aerodynamics [99] in the 1980s. The adjoint method has flourished for optimization problems in numerous disciplines, including geophysics [100], mechanical engineering [101] aerospace engineering [99, 102], computer graphics [103], and quantum mechanics [104, 105]. More recently, the adjoint method has been adopted for deep learning techniques in the form of backpropagation, which is discussed in detail in Section 6.4 [106], [107], [108], [109]. In the field of nanophotonics, the adjoint method was introduced around 2003 by Bendsoe et al. [101], and then has been intensively revisited as a promising design tool for various optical devices [43, 110].

**Figure 8:**
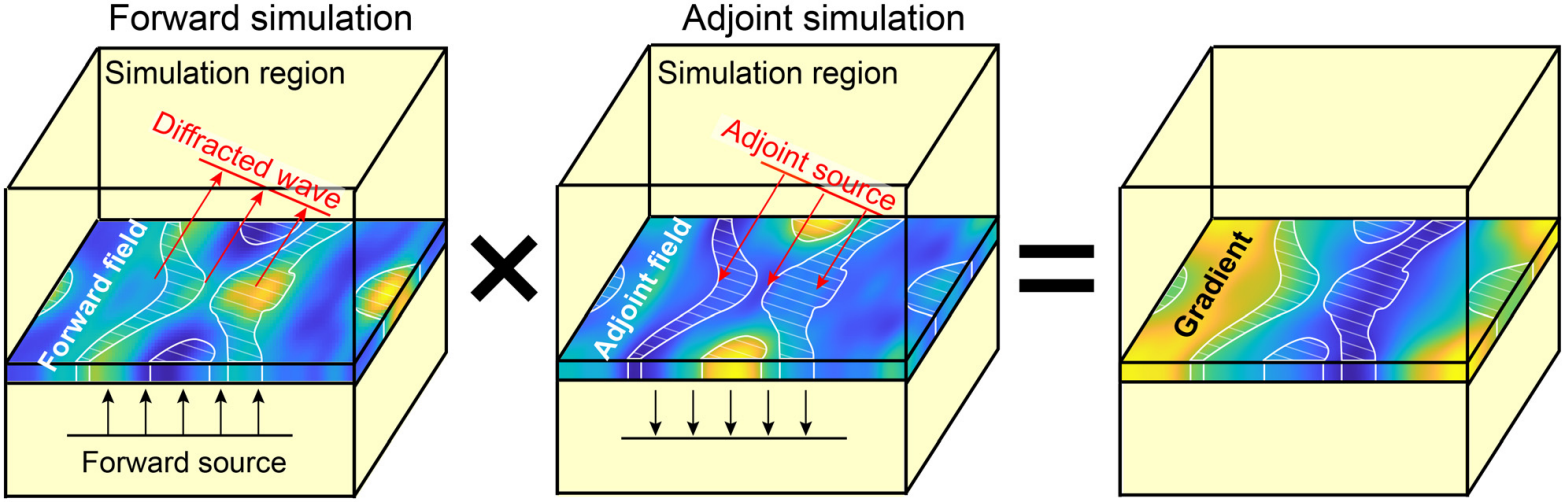
Conceptual image of adjoint method formulation for a beam deflector design problem. The simulation region is shown in half-transparent yellow. White shaded regions indicate regions filled with materials. Color contours represent the forward field (left), adjoint field (middle), and gradient distributions (right). (Left) In a forward simulation, a forward source (black arrows) is incident to evaluate FoM and forward field. A diffracted wave propagating toward the target direction is indicated as red arrows. (Middle) In an adjoint simulation, an adjoint source (red arrows) is incident to evaluate the adjoint field. (Right) FoM gradients are obtained through procedures including multiplication of the forward field and the adjoint field, providing guidelines on how material distribution should change to yield better FoM.

Unlike the finite difference method, the adjoint method can obtain the gradient of an objective function on all design parameters in only two simulations regardless of the number of design parameters, as schematically illustrated in Figure 8. This substantial reduction of the computation is possible by exploiting Lorentz reciprocity, which states that the relationship between an oscillating current source (e.g., an oscillating dipole) and the resulting electromagnetic field is unchanged if the source position and the point where the field is measured are interchanged [111]. Consequently, the effect of a material property (permittivity or permeability) perturbation in the entire design domain can be obtained at once by running an “adjoint” simulation where the current source is placed at the position of measurement in the “forward” simulation. The formulation of the adjoint-based method is more rigorously discussed in Section 5.1.

The adjoint method has been widely adopted in various photonic device optimizations including beam deflectors [41, 42], diffractive optical elements [112], metalenses [18, 39, 40], power splitters [43, 44], and wavelength demultiplexers [45]. The main advantage of the adjoint method is its fast convergence to local minima. Compared to evolutionary algorithms which typically require hundreds, often thousands of evaluations, the adjoint-based methods often converge in a few tens of evaluations. This is a compelling feature for those problems requiring computationally expensive electromagnetic simulations, such as 3D full-wave calculations using the FDTD method or the finite element method (FEM). A potential difficulty that may hinder a layperson from utilizing the adjoint method is that sufficient domain knowledge is required to derive an adjoint source analytically before actually running the adjoint simulations. This is not a trivial task for most design objectives, especially those regarding eigen frequencies [113], [114], [115].

### 5.1 Adjoint formulation

The formulation of the adjoint method can be built, starting from expressing Maxwell’s equation with Maxwell operator. Assuming a system of interest consists of linear materials, Maxwell’s equation can be expressed as follows:

(1) $$\begin{array}{l} Ax=b, \end{array}$$

where *A* is Maxwell operator with size *M* × *M* (*M* ≤ 3 is the dimension of electromagnetic field), **x** is a state variable (electric or magnetic field), and **b** is a source. Let’s define an objective function, *F*(**x**(**p**), **p**), as a function of state variables **x** and design parameters **p**. Our main interest is in calculating the derivative of objective function to the design parameter **p**:

(2) $$\begin{array}{l} \frac{dF}{dp}=\frac{\partial F}{\partial p}+\frac{\partial F}{\partial x}\frac{\partial x}{\partial p}. \end{array}$$

Assuming that *F*(**x**(**p**), **p**) is analytically known, ∂F/∂p and ∂F/∂x are easy to calculate. The main difficulty is in evaluating ∂x/∂p , which is typically implicit in the governing equation such as Eq. (1). In order to derive this, we first differentiate Eq. (1) to the design parameter **p**, resulting in:

(3) $$\begin{array}{l} A\frac{\partial x}{\partial p}=\frac{\partial b}{\partial p}-\frac{\partial A}{\partial p}x. \end{array}$$

The form of Eq. (3) is inherently same as Eq. (1), substituting **x** and **b** in Eq. (1) to *∂* **x**/*∂* **p** and ∂b/∂p−(∂A/∂p)x , respectively. This means that ∂**x**/∂**p** can be obtained using the same Maxwell’s equation, but applying different source terms. Using Eq. (3), if **p** is a vector of size *N*, to find complete *M* × *N* matrix *∂* **x**/*∂* **p**, one needs to solve *N* Maxwell’s equations for each different source column vectors, to obtain *∂* **x**/*∂p*_1_, *∂* **x**/*∂p*_2_, …, *∂* **x**/*∂p*_*N*_. Therefore, the total calculation time to get *dF*/*d* **p** would be proportional to the number of design parameters *N*. However, with some algebraical trick, we can reduce the number of equations required to obtain the gradient to only one. By multiplying arbitrary row vector *v* ^T^ to both sides of Eq. (3) yields

(4) $$\begin{array}{l} v^{\text{T}}A\frac{\partial x}{\partial p}=v^{\text{T}}\left(\frac{\partial b}{\partial p}-\frac{\partial A}{\partial p}x\right). \end{array}$$

Now if we set

(5) $$\begin{array}{l} v^{\text{T}}A=\frac{\partial F}{\partial x}, \end{array}$$

then Eq. (4) becomes

(6) $$\begin{array}{l} \frac{\partial F}{\partial x}\frac{\partial x}{\partial p}=v^{\text{T}}\left(\frac{\partial b}{\partial p}-\frac{\partial A}{\partial p}x\right). \end{array}$$

Note that the right-hand side of Eq. (6) consists of known quantities, except for *v* ^T^. *v* ^T^ can be obtained using transpose of Eq. (5), which is

(7) $$\begin{array}{l} A^{\text{T}}v={\left(\frac{\partial F}{\partial x}\right)}^{\text{T}}. \end{array}$$

Equation (7) is referred to as an adjoint problem. Since *F* is analytically known, the form of the right-hand side of Eq. (7) can be analytically found, and evaluated after solving the forward problem (Eq. (1)). By using *v* obtained from Eq. (7), Eq. (6) can be evaluated, leading to full derivative form Eq. (2). To summarize, only Eqs. (1) and (7) should be solved to evaluate the derivative *dF*/*d* **p**, regardless of the number of design parameters *N*. The process of obtaining the FoM gradient *dF*/*d* **p** based on the adjoint method is depicted in Figure 8. Note that one may derive the adjoint formulation using Lorentz reciprocity [41, 116], leading to the same result, which is trivial because Lorentz reciprocity is equivalent to the fact that Maxwell operator *A* is symmetric [117]. However, the symmetry condition is actually not needed, which will be explained in the paragraph below.

For Eq. (7), the form resembles Eq. (1), but the main difference is that the Maxwell operator *A* is transposed. Under time-harmonic assumption, since Maxwell operator *A* is equal to $\nabla \times {\bar{\bar{\mu }}}^{-1}\nabla \times -\omega^{2}\bar{\bar{\epsilon }}$ , the transpose of *A* corresponds to both ${\bar{\bar{\mu }}}^{\text{T}}$ and ${\bar{\bar{\epsilon }}}^{\text{T}}$ . Therefore, if related materials in the system are both symmetric, i.e. ${\bar{\bar{\mu }}}^{\text{T}}=\bar{\bar{\mu }}$ and ${\bar{\bar{\epsilon }}}^{\text{T}}=\bar{\bar{\epsilon }}$ , obviously *A* ^T^ = *A*. In this case, the system is identical to the forward problem (Eq. (1)). Even if they are non-symmetric, one may easily configure systems with *A* ^T^, by changing material properties using transposed permeability and permittivity matrices [116]. The previous arguments are also valid for systems including lossy materials, as the adjoint formulation does not require time-invariance property. Note that the arguments above still hold for general time-dependent systems, except that the inverse-time should be used for adjoint simulation [118]. For systems including nonlinear materials, the formulation above does not hold because we assumed a linear system in the first place. We will not discuss the adjoint method formulations for nonlinear systems here, but there is literature successfully addressing this case [119].

Since the FoM gradient obtained by the adjoint method is continuous, the updated design parameters will also have grayscale values. Therefore, one must apply specific penalization methods [120] to ensure the final optimized structure only exhibits binary features. Specifically, a continuation approach [121] has been suggested to avoid convergence to a bad local minimum, in which penalization factors gradually kick in. Assuming that the design space only allows two states (void and material), the position-dependent relative permittivity function can be expressed in $\epsilon_{r}=\epsilon_{1}+\zeta \left(\epsilon_{2}-\epsilon_{1}\right)$ , where $\epsilon_{1}$ means a relative permittivity of background material, $\epsilon_{2}$ means that of structure material, and ζ∈[0, 1] is a relaxation parameter. Binarization of the relative permittivity function can be realized via either penalizing the relaxation parameter [122] or applying filtering and regularization techniques [123, 124].

These days, the adjoint-based method works as a powerful tool for photonic optimization in combination with density filter methods [33, 39, 41, 125], level-set methods [126, 127], or neural network-based methods [128]. Among them, density filter and level-set based methods will be discussed in Section 7, in the context of robustness control against imperfections in the device fabrication process.

### 5.2 Free-form optimizations with adjoint-based method

**Figure 9:**
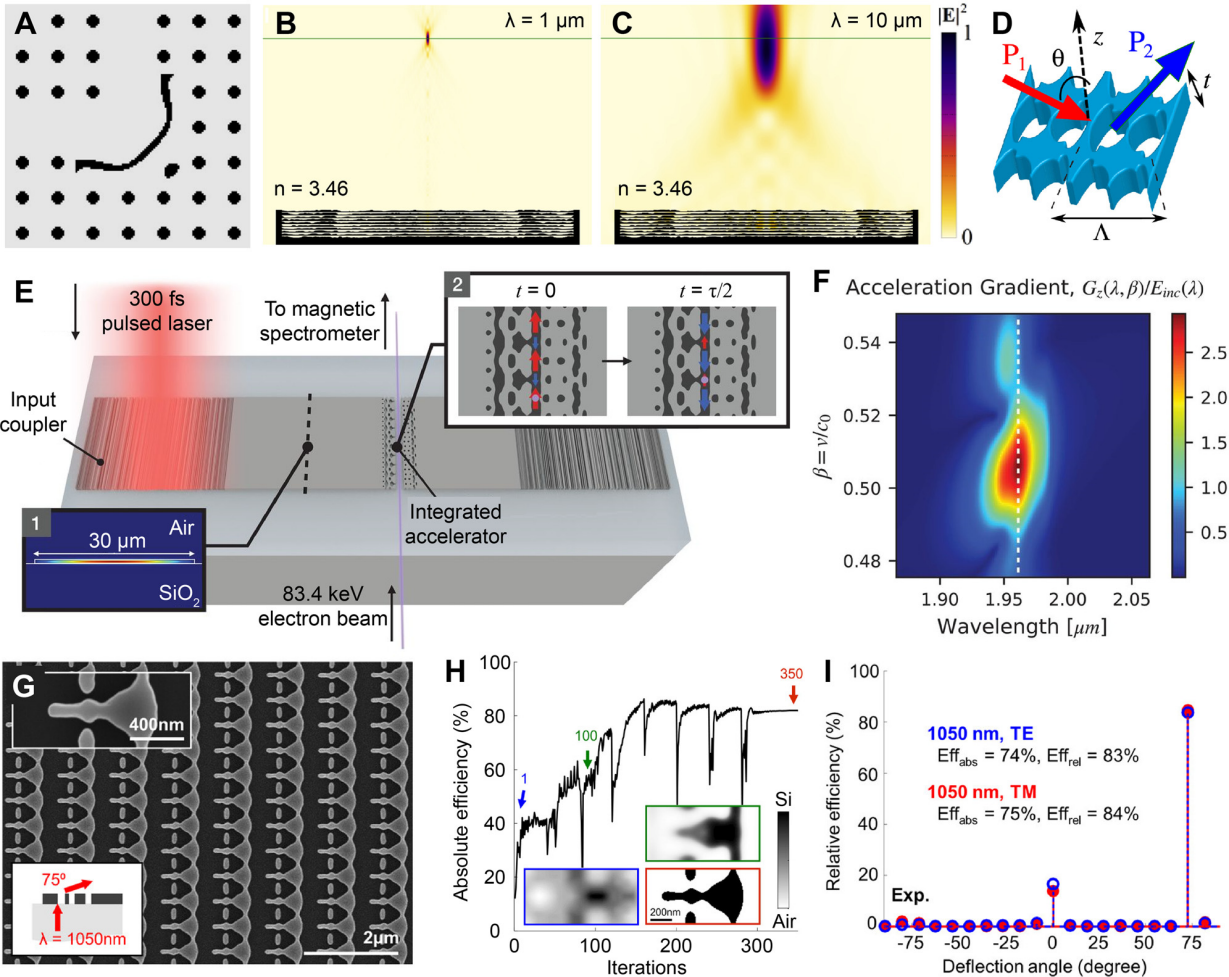
Photonic free-form optimization utilizing adjoint methods. (**A**) Image of optimized low-loss 90° waveguide bend structure. **A** Adapted with permission from [129]. Copyright 2004, AIP Publishing. (**B**–**C**) normalized electric field intensity plot of multiwavelength multilayer metalens with NA = 0.85, for wavelengths of 1 µm (**B**) and 10 µm (**C**), respectively. **B**, **C** adapted with permission from [131]. Copyright 2020. Optical Society of America (**D**) schematic of square lattice metasurface for second-harmonic generation. **D** Adapted with permission from [132]. Copyright 2018. Optical Society of America. (**E**) Schematic illustration of on-chip particle accelerator. The inset 1 shows the fundamental mode of a slab waveguide, coupled from normally incident free-space light via optimized grating coupler. The inset 2 shows two snapshots of the phase-matched fields and electron, separated by half an optical cycle ( τ /2). (**F**) Contour of normalized acceleration gradient as a function of operating wavelength and normalized input electron velocity. The dashed line indicates the optimal operation wavelength of 1.964 μm. **E**, **F** adapted with permission from [133]. Copyright 2020, AAAS. (**G**) SEM image of optimized polarization-independent 75° beam deflector for normally incident beam with 1050 nm wavelength. (**H**) Absolute efficiency evolution of beam deflector along optimization iterations. Inset surrounded by blue, green, and red boundaries represent permittivity distribution at iteration 1, 100, and 350, respectively. (**I**) relative diffraction efficiency for TE (blue circles) and TM (red circles) mode incident waves. **G**–**I** adapted with permission from [41]. Copyright 2017, American Chemical Society.

**Figure 10:**
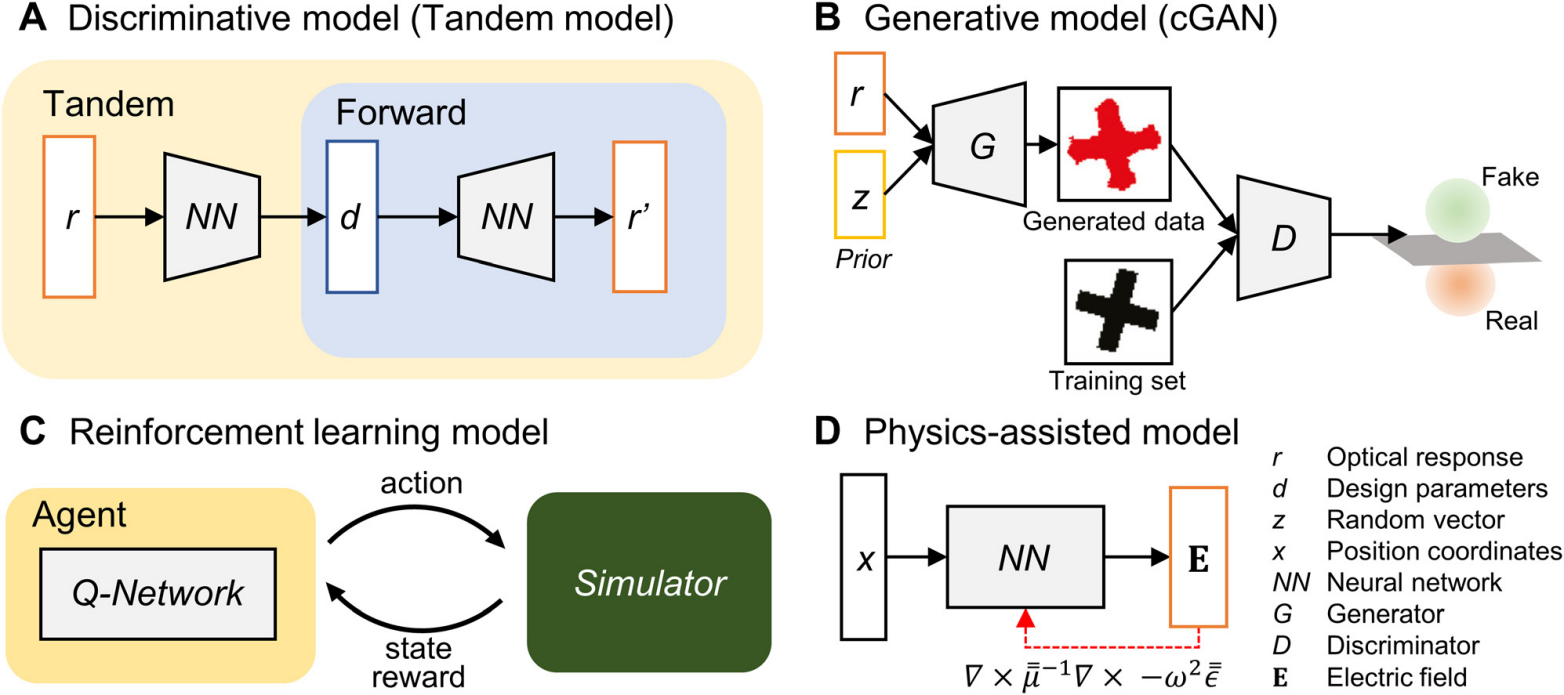
Schematic diagrams for each machine learning model used in nanophotonic device designs. (**A**) Tandem network is one type of discriminative model that is widely used for inverse-design of a device. (**B**) The figure depicts a conditional GAN (cGAN) architecture which takes the required optical conditions as an input and generates a two-dimensional design geometry. **B** Adapted with permission from [36]. Licensed under CC BY 4.0. (**C**) A schematic diagram of a reinforcement learning model involving a Q-network. The iteratively acquired knowledge of the system from the simulator is accumulated in the Q-network of the agent. (**D**) A physics assisted model returning the electric field at a given point is depicted. The network is regulated by the physics equation governing the system.

The early works of Jensen et al. [129] (Figure 9A), Burger et al. [130], and Miller et al. [116] provided the theoretical background and mathematical formulations of the adjoint method in application to Maxwell’s equations. Then, impactful works have been followed by Christiansen et al. [131], Sitawarin et al. [132], Sapra et al. [133], and Sell et al. [41]. The work by Christiansen et al. demonstrated axisymmetric, multi-wavelength, multi-layer metalenses via the adjoint method [131]. The radial distribution of each axisymmetric layer of multi-layer metalens was optimized in a free-form manner. Their work proposed reconfigurable, widely separated multiwavelength lenses which can be focused at *λ* = 1 µm (Figure 9B) and 10 µm (Figure 9C). Regarding multifunctional beam focusing devices, the works by Lin et al. [134] and Camayd-Munoz et al. [135] are also notable. Lin et al. [134] demonstrated multifunctional metalenses by optimizing each lateral distribution of five layers in a 2D multilayered system, yielding angular aberration correction and angle-convergence features. The angular aberration-corrected metalens and the angle-convergent metalens showed diffraction-limited focusing with transmission efficiency above 25 and 15%, for incident angles ranging from −20° to 20° and −9° to 9°, respectively. Camayd-Munoz et al. [135] designed a multifunctional spectral filter for image sensors. The authors first optimized the 3D material distribution of polymer cube, directing incident light to desired RGB sensor location depending on wavelengths and polarizations. As a nanofabrication-compatible option, the authors also suggested a 3D spectral filter composed of five discrete layers, which can be fabricated by multi-layer lithography. The five-layer stacked volumetric spectral filter outperformed conventional absorptive filters in terms of sorting efficiency (57%), color contrast (29%), and polarization contrast (41%). In nonlinear optics, Sitawarin et al. [132] discovered free-form micro-structured fibers and metasurfaces via adjoint optimization for nonlinear frequency-conversion. In their work, the cross-section of fiber was inverse-designed for third-harmonic generation, and the unit-cell of square lattice metasurface (Figure 9D) was optimized for second-harmonic generation. Their inverse-designed third-harmonic generation fiber showed 10^4^ times enhanced pump power requirement compared to plain silica fibers [136], and inverse-designed metasurface showed 10^7^ times enhanced second-harmonic generation conversion efficiency compared to a manually designed device [137]. For arbitrary polarization control of light, Shi et al. [138] proposed a free-form metasurface having continuous angle-tunable birefringence. The unit-cell of periodic metasurface was optimized in a free-form manner to yield elliptical birefringence, which cannot be achieved from mere material anisotropy or regularly shaped, hand-designed structures. Specifically, the metasurface was designed so that the horizontal linear polarized incident light is converted into right circular polarization, horizontal linear polarization, and 45° linear polarization at the angle of incidence −60°, 0°, and 60°, respectively. The fabricated metasurface showed an experimentally measured degree of circular polarization of 0.94 at −60°, and the degree of linear polarization of 0.99 and 0.96 at 0° and 60°, respectively.

The adjoint-based optimization also significantly contributed to the design of silicon photonic devices. Recently, Sapra et al. [133] designed an on-chip integrated particle accelerator driven by a laser as shown in Figure 9E. In their work, two devices, the grating coupler converting free-space Gaussian laser pulse to the fundamental TE mode in a slab waveguide shown in the inset 1 in Figure 9E, and the integrated metagrating structure (the inset 2 in Figure 9E) exhibiting the desired electric field distribution to accelerate incident 83.4 keV electron beam were optimized via the adjoint method. The spectrum of normalized acceleration gradient for the cascaded system of the optimized grating coupler and accelerator is shown in Figure 9F. The dashed line indicates the peak operating wavelength of 1.964 μm, which slightly deviates from the target wavelength 2 μm due to finite device length and numerical dispersion. The optimized particle accelerating system experimentally showed a maximum acceleration gradient of 30.5 MeV/m, inferred from particle-tracking simulations. Vercruysse et al. [139] demonstrated the application of a slow-light optical-phased array based on photonic crystal waveguides. The two-stage mode converter was optimized using the adjoint method so that the fundamental TE mode is maximally coupled to photonic crystal waveguides. The dispersion and radiative loss of the photonic crystal waveguide were engineered in a free-form manner to steer the input wave into wavelength-dependent target directions efficiently. The beam steering was achieved for a 20° steering range within a 20-nm bandwidth. One of the pioneering works in beam deflection includes Sell et al. [41] who designed large-angle, multifunctional metagratings. The polarization-independent beam deflector as shown in Figure 9G and the wavelength-dependent beam splitter was proposed. For both devices, the FoM was set to the diffraction efficiency along desired target directions. The metagratings were allowed to have arbitrary shape during optimization. As the optimization continues, the grayscale permittivity distribution is gradually binary-pushed, as shown in the insets of Figure 9H. As a result, the beam deflector was experimentally demonstrated to have a 75° deflection with a relative diffraction efficiency of over 80% for a normally incident wave at 1050 nm regardless of the polarization direction (Figure 9I). The beam splitter showed 82 and 77% relative diffraction efficiencies for 1000 and 1300 nm incident TE waves, respectively. These results are far superior to conventional design approaches (e.g., effective medium [140], transmit array [141], geometric phase [142] and echelle grating [140]) in terms of both deflection angles and efficiency.

As discussed above, the adjoint-based inverse design has discovered many revolutionary photonic devices. However, since the optimization algorithms using adjoint method are inherently gradient-based, the optimization sometimes converges to a poor local optimum, depending on its initial guess of the design parameters. To avoid this issue, one may benefit from advanced gradient-based optimizers, such as sequential least squares programming (SLSQP) [143] and method of moving asymptotes (MMA) [144]. Also, as described in Section 4.3, one may run multiple adjoint optimizations using “cold-start” or “warm-start” strategies. Consequently, these efforts eventually add up the number of simulations required for achieving high-performance designs. As an alternative solution, researchers have recently been trying to combine the adjoint-based optimization method with machine learning algorithms, which is discussed in detail in Section 6.4.

Another reason adjoint optimization converges to poor local optima is a penalization method that enforces a material status to have zero (void) or one (material). Without any penalization, adjoint optimization generally converges to a relatively good optimum with grayscale material with a material status value between zero and one [145]. Thus, studying efficient penalization methods, which have been studied for decades in mechanical engineering [121, 123], may address this issue in photonic adjoint optimization.

## 6 Machine-learning-based methods

Recent developments of deep-model-based machine learning have revolutionized many scientific research fields, from pattern recognition [146, 147] to image creation [148], from speech recognition [149, 150] to language translation [151], and from the game of Go [152] to protein folding [153]. The field of nanophotonics is no exception. Machine learning has provided solutions to the device design optimization problems in nanophotonics that were previously impractical to solve due to their complex and large search spaces. To this end, in this section, we review the works that tackle nanophotonics free-form optimization problems using machine learning techniques. As illustrated in Figure 10, we classify those works into four branches: discriminative, generative, reinforcement learning, and physics-assisted. A discriminative model aims to learn a map from device design to the optical response from the pre-simulated data (Figure 10A). A generative model aims to mimic the data distribution in the training set by learning to generate data that is indistinguishable from the training data (Figure 10B). In the reinforcement learning framework, the artificial intelligence, often termed an “agent”, explores through the problem space by iteratively making decisions and learning from its outcomes (Figure 10C). Unlike the three models where the model training is entirely data-driven, a physics-assisted model puts constraints on the output of a model according to the physical laws that govern the given system (Figure 10D). We introduce each methodology in detail and provide previous works that accomplished free-form optimizations with the corresponding method in the following subsections. As a side note, multiple review articles have been published on the similar topic with different emphases: Jiang et al. [154] and Ma et al. [155] provided a thorough introduction to applying deep neural networks in the design problems of photonic devices, and So et al. [156] focused on the inverse design using machine learning in which the reinforcement learning is also included. We would like to invite the readers who find this topic intriguing to take a further look into those review papers.

### 6.1 Discriminative model

A discriminative model utilizes a neural network to catch the nonlinear relationship between the input and the output through a data-driven training process. The most elementary form of an artificial neural network is a fully connected neural network consisting of stacked nonlinear layers, where each layer is a nonlinear transformation of the form $\sigma \left(W^{\text{T}}x+b\right)$ . Here, **x** is the input vector to the network, or the output vector of the previous layer, *W* is the weight matrix, **b** is the bias vector, and *σ* is an activation function. Hyperbolic tangent, sigmoid, and rectified linear unit [157] are examples of widely used activation functions. A typical neural network is trained through a repeated process of backpropagation in which the elements of the weight matrices are adjusted to minimize a given loss function that is defined as a function of the final output of the network [107]. The implementation and training of neural networks can be conveniently done by using open source deep learning frameworks such as PyTorch [158] and TensorFlow [159].

**Figure 11:**
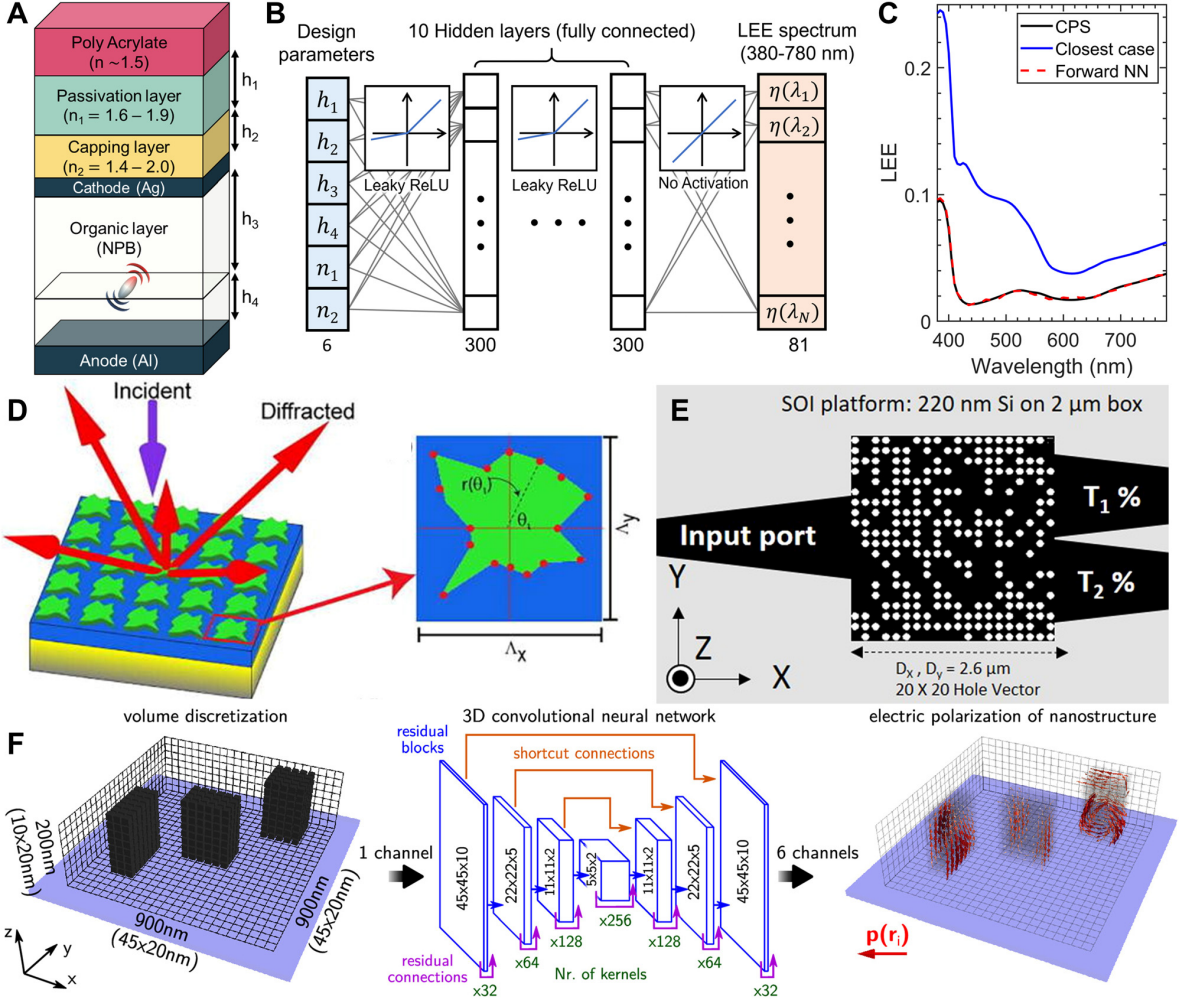
Use of discriminative neural networks in nanophotonics. (**A**) A schematic of simplified top-emitting OLED structure. (**B**) A diagram of the forward neural network structure. Ten hidden layers, each with 300 nodes constitute the neural network. Leaky rectified linear activation (Leaky ReLU) function is used as activation functions. (**C**) The LEE curve calculated from the CPS model (black solid line), forward neural network (red dashed line). The LEE curve of the closest sample in the training dataset is plotted together (blue solid line). **A**–**C** Adapted with permission from [161]. Licensed under CC BY 4.0. (**D**) A meta-atom geometry defined by a sixteen-sided polygon. The vertices are defined by polar-coordinates (*r*, *nπ*/2). **D** Adapted with permission from [166]. Copyright 2018, AIP Publishing. (**E**) A schematic of an inverse-designed integrated photonic power splitter. Pixels marked by a white circle are in the etched state (*n* = *n*_silicon_), and the remaining black pixels are the unetched pixel (*n* = *n*_silica_). **E** Adapted with permission from [167]. Licensed under CC BY 4.0. (**F**) Prediction of the field distribution using a neural network. U-net like neural network architecture is used for the retrieval of data in the same spatial domain. **F** adapted with permission from [168]. Copyright 2020, American Chemical Society.

Peurifoy et al. was one of the first to notice the potential of employing machine learning to nanophotonics [160]. They accomplished the inverse design of a multilayer concentric spherical shell using a fully connected neural network, which accepts the layer thicknesses of the multilayer shells as the input and predicts the spectrum of scattering cross section sampled at multiple wavelength points. The trained neural network accurately predicted the scattering cross section spectra even for the designs that were not shown during the training. This study provides an excellent example of how neural networks can be used to design nanophotonic devices. Kim et al. [161] utilized a similar neural network architecture to predict the light extraction efficiency of a planar organic light emitting diode (OLED) as illustrated in Figure 11A–C. The thickness and refractive indices were used as the inputs to the fully connected network which predicts the light extraction efficiency spectrum (red dashed line in Figure 11C) with root-mean-squared-error of 1.86 × 10^−3^, compared to the ground truth values generated by rigorous electromagnetic calculations based on Chance–Prock–Silbey (CPS) model (black solid line in Figure 11C) [162].

One advantage of a discriminative model compared to previous methods is that it can conduct diverse optimization tasks once a network is trained. In their work on OLED design [161], Kim et al. combined the neural network with GA to tackle multiple optimization problems. Since the inference time of the network is ∼10^3^ times faster than the rigorous solver, the evolutionary optimizer is able to consider a huge number of candidates for finding optimal designs. Such optimization problems include non-trivial tasks including an inverse design of OLED device structures using light extraction efficiency spectrum, and the design of OLED that has minimal angular color difference. Many other studies were also carried out using a fully connected neural network [27, 163], [164], [165]. However, most of these design problems have a DoF between 2 and 10 and the shape is fixed to a primitive geometry, which is far from a free-form optimization.

Nanophotonic structures with non-trivial shapes have also been designed using discriminative models. Inampudi et al. [166] designed high-efficiency metagratings by utilizing the neural network as a fast predictor that replaces the computationally heavy Maxwell solver. The unit cell of the metagratings contains a sixteen-sided polygon as shown in Figure 11D, which is characterized by the distances between its vertices and the origin. The polar angle between each pair of the neighboring points is fixed to 2*π*/16. The proposed neural network receives the radial components of the vertices of the polygon as the input and predicts the diffraction efficiency as the output.

To cover a wider domain in the design space as a free-form design, it is required to go beyond employing non-primitive shapes and lift the constraints on the device topology. Tahersima et al*.* [167] demonstrated the inverse design of integrated photonic power splitters using a two-dimensional binary representation as depicted in Figure 11E. The binary image represents the etched-state of the points inside the square power splitter which are meshed into 20 × 20 square lattice points. To predict a power flowing into each port of the power splitter, Tahersima et al. trained a forward neural network that takes the 400 points representing the device as inputs and predicts the transmission spectrum at each terminal. Also, an inverse neural network was created to solve the inverse problem of obtaining the power splitter geometry from transmission and reflection spectra. It should be noted that the dataset of power splitter designs used to train the neural network were not from random sampling, but were the result of heuristic optimizations. Hence, an additional computational power was spent in the data preparation step compared to random sampling.

The input size of a neural network is normally fixed, hence finding a good representation that can cover the full complexity of the entire design space while keeping the number of dimensions small is a nontrivial task. Methods for the reduction of dimensionality of the representation discussed in Section 3 are not guaranteed to work for every optimization task. Instead of searching for an adequate representation to cover the free-form design space, we can let the model discover a relevant set of features that serves as a nonlinear basis of the representation, from a set of two-dimensional input images using a convolutional neural network (CNN). A CNN is a network architecture that is widely used for image processing. A convolution layer of a CNN extracts features from an image using kernels. Based on the assumption that neighboring pixels are correlated, a set of kernels works as a filter that sweeps the 2D image and collect the convoluted values. Unlike the fully connected network where every possible pair between the neighboring layers has an individual weight assigned to it, the trainable parameter of a convolution layer is limited to the number of elements in a kernel, which can be as small as 3 × 3 per channel. A small number of trainable parameters in CNN implies that the features of a 2D image can be extracted with a network of smaller capacity compared to a fully connected one.

Wiecha et al. [168] utilized CNNs to predict the electromagnetic field distribution inside a simulation space with a given nanophotonics structure as illustrated in Figure 11F. The design space was limited to cuboids in case of silicon nanopillars, where a polygon with holes was used in case of planar gold nanostructures. Here, the authors used the coupled dipole approximation (CDA) [169], [170], [171], [172], [173] that approximates every cell inside the nanostructure as an oscillating dipole moment and retrieves the near-field and far-field field distributions correspondingly. An U-net like architecture was adopted as the neural network design [174]; a combination of convolutional layers and max pooling layers is used to extract the features from the design space (3D image of structure) and the dipole distribution at each mesh is retrieved through convolutional layers and upsampling layers. Shortcut connections maintain the spatial information during the contracting and expanding paths, and residual connections enable the gradients to flow through and between layers more easily. These connections are known to be useful for maintaining a good learning performance on a deep architecture. Two different neural networks were trained for field prediction. One neural network was for planar gold polygons and the other was for prediction of silicon cuboid blocks. With the prediction from the trained neural networks, the authors were able to calculate the derived values such as the energy flux or the scattering direction in the far-field. Furthermore, phenomena such as the anapole mode [175], [176], [177] in silicon cuboids or near-field interaction between the silicon cuboid dimers situated between a nanogap were observed. This work is distinguished from other works in a sense that the network is able to retrieve the field distribution inside the simulation space, thereby enabling access to all kinds of derived phenomena.

In the inverse design of nanophotonics devices, one of the major obstacles that researchers face is the problem of one-to-many mapping between the optical response and design parameters (i.e., multiple device structures result in the identical target optical response). If a discriminative neural network is designed to have an optical response as the input and retrieve the design parameters as the output, the data-driven training process would require the uniqueness of the design parameters for the loss to be well-defined. The one-to-many mapping problem can be avoided by providing additional optical responses as the design target and lifting the degeneracy [161] but this is limited to cases where the additional optical response can be provided. Tandem network is one of the first approaches that circumvents the issue through alternative network design [27, 163], [164], [165]. In a tandem configuration, an inverse neural network is followed by a pre-trained forward neural network, which maps the design parameters to optical response space as schematically shown in Figure 10A. The joint network is trained so that the input and the output of the network are the same, while the weights of the forward neural network are fixed. By retrieving the parameters at the beginning of the forward neural network, the inverse design is accomplished. A mixture density network is another type of architecture for inverse design where the output is the parameters for probability distributions, in which the design parameters are sampled [178]. The one-to-many mapping issue becomes more prevalent as the complexity of the design space is increased. Hence in the next subsection we introduce generative networks that generate design parameters from a target optical response concatenated with a randomly sampled latent variable. Inclusion of the latent variable enables the neural network to distinguish different device structures that are mapped to the same optical response.

### 6.2 Generative model

Discriminative models are found to be useful for optimization tasks in parametric design spaces. However, the scheme easily fails in the large design space such as 2D grid optimizations, due to insufficient training data compared with its vast search space. To address such problems, utilization of generative models was introduced. Generative models learn the underlying pattern in the training set which consists of samples with high FoM value and generate data in the domain space based on the observed pattern. Once the pattern is learned, the network can generate multiple patterns with required optical characteristics. In this subsection, we will discuss the autoencoder-based architectures and multiple variations of the generative adversarial network (GAN) as examples of their application in the field of nanophotonics.

#### 6.2.1 Variational autoencoder

**Figure 12:**
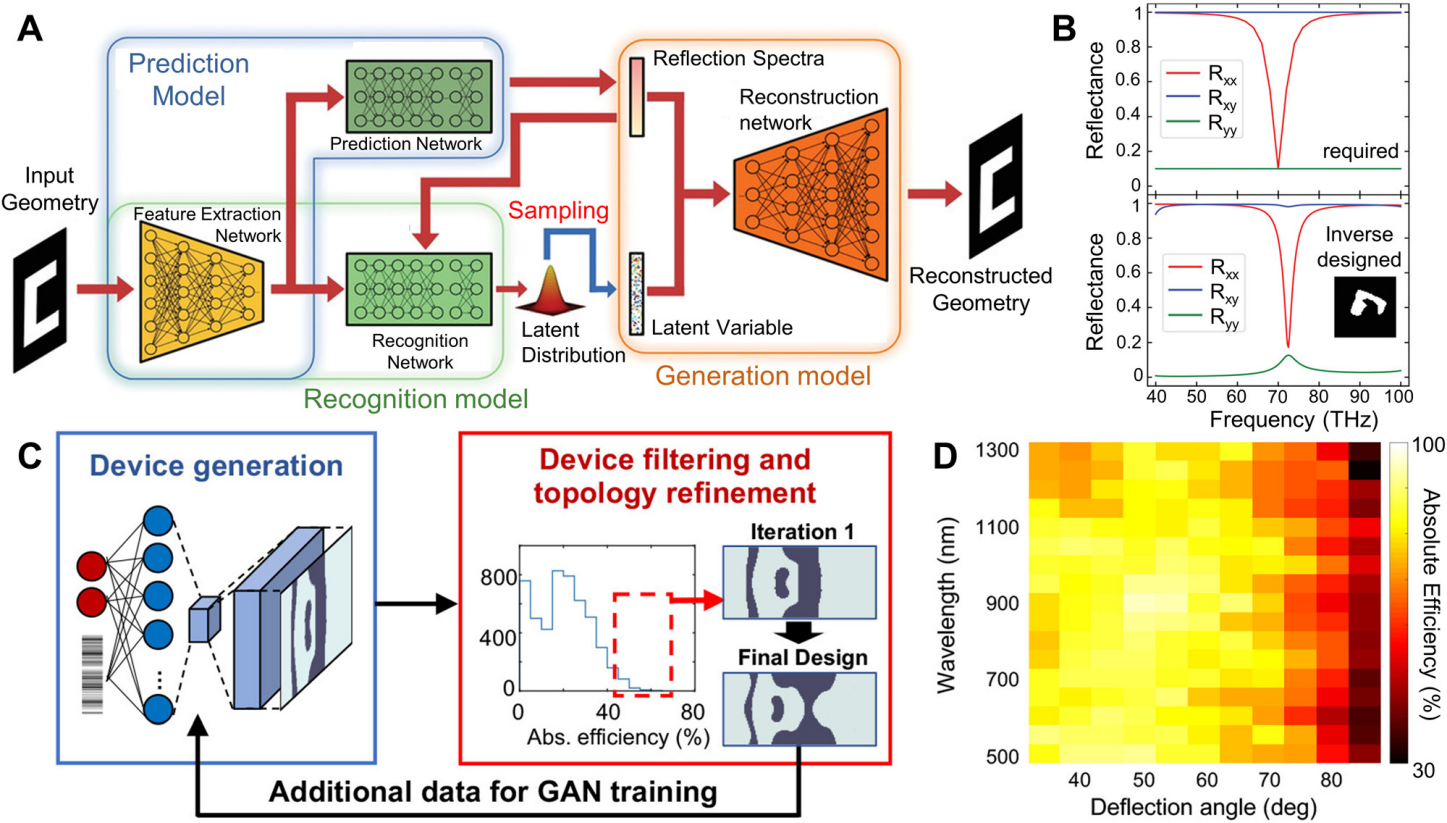
Use of generative models for the design of nanophotonic devices. Most generative models generate a binary image of the nanostructure, and thus removes the necessity of parametrizing the design geometry. (**A**) A schematic of variational autoencoder used in the inverse design of reflectance spectrum. Reflectance spectrum and latent parameters are used to generate a device geometry. (**B**) Generated nanostructure successfully inverse designs the target reflection spectrum. Subfigures A and B were adapted from Ma et al. **A**, **B** adapted from permission from [180]. Copyright 2019, Wiley. (**C**) Designing a two-dimensional beam deflector using a conditional generative adversarial network and an adjoint-based optimization. Two red circles in the device generation section denote the condition variables (wavelength, deflection angle) provided to the generator. (**D**) Absolute efficiency of the two-dimensional beam deflector for various conditions. It displays of the highest device efficiencies for metagratings generated by the GAN generator and then fine-tuned by adjoint methods **C**, **D** adapted with permission from [35]. Copyright 2019, American Chemical Society.

Variational autoencoder (VAE) is one type of generative model which has a network structure based on the autoencoder architecture [179]. Use of VAE in photonics assumes that a device structure and the corresponding optical response can be jointly encoded into a latent space where each of the latent variables follow a certain type of probabilistic distribution. A typical VAE can be decomposed into two networks as shown in Figure 12A: the anterior part is an encoding network which extracts the important features of the device design and maps to the latent space; the posterior part is the decoding network which admits variables sampled from latent distribution and maps into the design space. Use of latent variables in the generator enables the neural network to distinguish different device structures that are mapped to the same optical response. It should be noted that the network architecture depicted in Figure 12A is slightly different from the original VAE, as it contains the prediction model.

Ma et al. used VAE in the inverse design of a two-dimensional free-form metasurface [180]. They inverse designed a free-form metal resonator device with given polarization-dependent reflectance spectra. Three models – a prediction model, a recognition model, and a reconstruction model – were involved in the inverse design as schematically illustrated in Figure 12A. The prediction model admits a 2D image of the input geometry and predicts the polarization-dependent spectra as the output. The recognition model maps the information of the input geometry and the reflection spectra to the latent space. The prediction model and the recognition model both share a convolutional neural network which reduces the dimensionality of the data before entering the fully connected layers. Latent variables, which are sampled from the latent space distribution and reflection spectra for inverse design, enter the reconstruction network consisting of deconvolution layers to reconstruct the device structure. The entire network was trained in an end-to-end manner with the network loss defined as a weighted sum of generative and deterministic loss. With such a model, the inverse design of the polarization-resolved reflection spectra was successfully done as depicted in Figure 12B.

#### 6.2.2 Generative adversarial network

Generative adversarial network (GAN) is another type of generative model in which the neural network tries to mimic the data distribution in the training set by running a minimax game between a generator and discriminator [181], [182], [183], [184]. As depicted in Figure 10B, generator network accepts a random variable as an input and tries to create a fake data that resembles the true data distribution in the training set. On the other hand, the discriminator tries to distinguish between the generated data and the original data in the training set. By running a minmax game between the generator and discriminator, the generator is trained to mimic the training data distribution. GAN has proven to be successful in a wide range of applications including image creation, image-to-image translation, and image synthesis, where diverse variations of GAN have been designed accordingly [148, 149, 185], [186], [187], [188], [189], [190]. GAN has also been implemented in the field of free-form nanophotonic optimization. Among various network structures, conditional GAN (cGAN), whose structure is depicted in Figure 12C, is one of the most widely used network structures in the field of photonics [186]. Unlike the original GAN, a cGAN accepts additional parameters related to the target design as an input to the network. Jiang et al. [180] trained a cGAN network that designs a two-dimensional metasurface beam deflector. The network was designed to receive the operating wavelength and the deflection angle as inputs and returns a 2D image of the metagrating with a high deflection efficiency as an output as shown in Figure 12C. The cGAN network is followed by an adjoint-based optimization step and the resulting data was added to the training set for the network training. As can be inferred in Figure 12D, the network designed metagratings with high efficiency in a wide range of target wavelength and deflection angle. Note that, however, optimization through this method requires a training set containing highly performing device designs.

Despite its successful utilization in device designs [35], [36], [37], the training process of GAN is known to face problems such as vanishing gradient [191], mode collapse [192], and an absence of well-defined similarity metric [192]. In particular, a conventional GAN often fails to converge when the support of the training data distribution has little or no overlap with that of the generated data. Wasserstein GAN (wGAN) was introduced to address these problems, which replaced Kullback–Leibler divergence with Wasserstein distance as a similarity measure [192]. The Wasserstein distance, otherwise known as earth mover’s distance, is defined as the minimum cost needed to transfer one probability distribution to the other. Since the Wasserstein distance is well-defined even when the supports of the two distributions are non-overlapping, wGANs can be robustly trained and are more stable compared to conventional GANs [191, 192].

An et al. applied wGAN to design free-form meta-atoms [38]. In their work, a generator is designed to accept a complex transmission amplitude spectrum with a random noise vector and returns a two-dimensional image of the metasurface, while the discriminator was designed to distinguish between real and fake samples based on the two-dimensional image and its corresponding spectrum using the Wasserstein distance. Along with this neural network architecture that combines cGAN and wGAN, an auxiliary network that predicts the complex transmission amplitude spectrum from a given structure was trained. Using wGAN and the auxiliary network, the authors generated a library of meta-atoms with desired characteristics and designed a variety of multifunctional metasurfaces including a bifocal metalens, a polarization-multiplexed beam deflector/metalens, and a polarization-independent metalens by assembling the metaatoms.

### 6.3 Reinforcement learning

Along with the rise of deep learning methods, reinforcement learning (RL), a branch in optimization methods, has also gained much attention with improved performance utilizing the neural networks. To date, a few studies have applied RL in various optimization problems in nanophotonics [193], [194], [195], [196]. In this subsection, we introduce the basics of RL and the procedures to frame optimization problems in nanophotonics as RL problems. Particularly, we describe the commonly used RL framework in nanophotonics, namely the Deep Q-Network (DQN) [197]. It should be noted that optimizations with RL does not utilize a pre-computed dataset but requires large computing resources for training DQN. The nanophotonic optimization examples using q-learning and actor-critic framework will be discussed.

As its name suggests, RL is a machine learning framework that learns from experiences of repeated trial and error. In order to frame the trial and error, two major components must be defined: an environment and an agent. The environment includes the dynamic models and provides measures describing the current status that an agent is in. The agent reads the information provided by the environment and generates actions that can change the state of the environment. Through the repeated interaction between the agent and the environment, which is schematically illustrated in Figure 10C, the agent is able to change the environment in order to maximize the rewards collected. The whole process is formally defined as a Markov decision process (MDP), which is described using five tuples: (*S*, *A*, *R*, *P*, γ). *S* is the space in which a state of the environment is defined, $s,s^{\prime }\in S$ . *A* is the space in which the action of an agent is defined, a∈A . *R* is the reward function given a state and an action, *r* = *R*(*s*, *a*) and *P* is the transition probability to a next state $s^{\prime }$ given the current state *s* and action *a*, $P\left(s,\;s^{\prime }\right)=\text{Pr}\left(s_{t+1}=s^{\prime }|s_{t}=s,\;a_{t}=a\right)$ . Lastly, *γ* is the discount factor for discounting the temporally distant reward values.

The main objective of RL is to maximize the expectation of collected reward, or return. Formally the objective is defined as

(8) $$\begin{array}{l} \text{maximize }E_{\pi }\left[\sum_{i}\gamma^{i}R\right], \end{array}$$

where π(*a*|*s*) is a policy which defines a probability distribution over action a∈A given the state s∈S . Assuming that the agent follows this policy, a *value function*, which is the expected return given a state, is defined:

(9) $$\begin{array}{l} v_{\pi }\left(s\right)=E_{\pi }\left[\sum_{i}\gamma^{i}R|s_{t}=s\right]. \end{array}$$

Similarly, the value function given state *s* and an action *a*, known as *state-action value function*, is defined as follows

(10) $$\begin{array}{l} q_{\pi }\left(s,a\right)=E_{\pi }\left[\sum_{i}\gamma^{i}R|s_{t}=s,a_{t}=a\right]. \end{array}$$

Utilizing these functions, the objective of RL can be put as finding the optimal state-action value function such that

(11) $$\begin{array}{l} q_{*}\left(s,a\right)=\max_{\pi }q_{\pi }\left(s,a\right). \end{array}$$

Learning the optimal state-action value is done through a method of dynamic programming called Bellman optimality equation [198] which states

(12) $$\begin{array}{l} q_{*}\left(s,a\right)=R\left(s,a\right)+\gamma \max_{a^{\prime }}q_{*}\left(s,a^{\prime }\right). \end{array}$$

We denote *q*(*s*, *a*) as the approximation to q_*_(*s*, *a*). By iteratively applying the gradient descent on the state-action value function using the error from the Bellman equation yields an algorithm called q-learning [199]:

(13) $$\begin{array}{l} q\left(s,a\right)\leftarrow q\left(s,a\right)-\alpha \left(R\left(s,a\right)+\gamma \max_{a^{\prime }}q\left(s,a\right)-q\left(s,a\right)\right). \end{array}$$

With the aid of deep neural networks, even the complex state-action value function *q*_*π*_(*s*, *a*) and the policy function *π*(*a*|*s*) of RL algorithms can be approximated. This combination of RL algorithms and deep neural networks opened a field of deep reinforcement learning.

**Figure 13:**
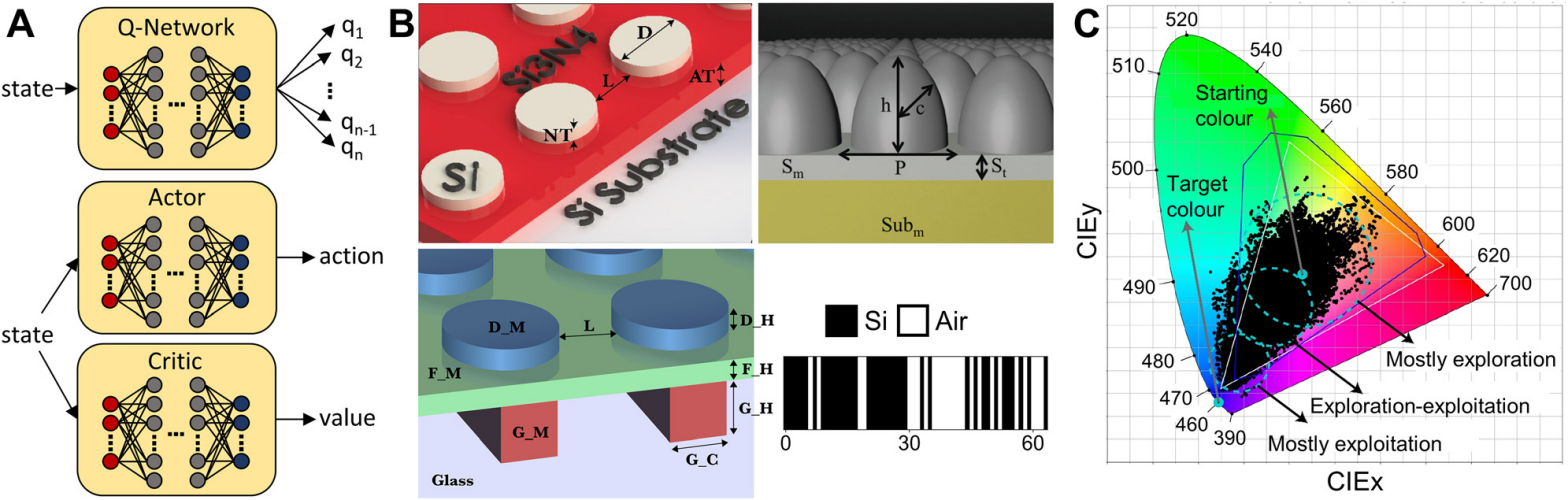
Use of reinforcement learning frameworks in nanophotonic device designs. (**A**) Two most widely used frameworks of deep RL. The top figure is value-based q-learning which uses a neural network to approximate the q function. This structure is suitable for discrete finite action space and chooses action according to the *q* values of the state-action pairs. The bottom figure represents the actor-critic framework in which two different neural networks are used to approximate an actor and a critic. Critic approximates the state value or the state-action value as the q-network. On the other hand, the actor approximates a policy, which directly maps the state to action or a probability distribution over the action space. (**B**) Different parameterizations of the devices optimized in each of the RL methods [193], [194], [195], [196]. **B** (Up-left) adapted with permission from [193]. Copyright 2019, Optical Society of America. **B** (Up-right): Adapted with permission from [195]. Copyright 2020, Royal Society of chemistry. **B** (Down-left) adapted with permission from [194]. Licensed under CC BY 4.0. (Down-right) Adapted with permission from [196]. Licensed under CC BY 4.0. (**C**) A progression of optimization from Ref. [193] that shows how an RL agent typically behaves during optimization. Initially, the agent explores, covering a large search space. As the learning progresses, the agent explores less and exploits more, converging to the optimum. **C** adapted with permission from [193]. Copyright 2019, Optical Society of America.

One exemplary work in deep RL is DQN, which uses deep neural networks as function approximators to the state-action value function, also known as the q-network whose schematic illustration can be found in the upper panel of Figure 13A. Several works in nanophotonic optimization have adopted DQN as an agent to design the nanostructures [193], [194], [195], [196]. Sajedian et al. [193] optimized color generation by dielectric nanostructures and found designs that are much closer to the true colors compared to those found by the human researchers. In the same year, Sajedian et al. [194] optimized device structure for metasurface holograms, achieving the computed transmission efficiency of 32% for high-quality holograms, which is superior than the previously reported results under the same conditions. Lastly, Badloe et al. [195] optimized a design of ultra-broadband absorbers, finding designs with absorptions over 90% for various materials. While all works address different problems, the way the problems are set up closely resembles one another.

The three works [193], [194], [195] share a common framework of optimizing a device structure. First, the state is defined with a fixed number of geometrical parameters of pre-defined topological structures. The detailed parameters can be found in Figure 13B. The reward is given by respective FoM that are calculated from simulated environments, using the state as the input. The action is given by the choice of increase or decrease on one of the state variables, by a predefined constant. When the action is applied, the state changes by the amount chosen by the action, on the variable which is also chosen by the action. Under these definitions, the DQN agent operates by repeated process of trial-and-error, optimizing the q-function. Specifically, the agent starts with an initial state corresponding to the parameters of the device at the start of optimization. Then, the agent applies the action, changing the state variables, for a given number of steps and collects and stores transition data from each step to a memory structure called experience replay buffer. The transition data consists of five tuples, (*s*_*t*_, *a*_*t*_, *r*_*t*_, *d*_*t*_, *s*_*t*+1_). Here, *s*_*t*_ is the state at which the action *a*_*t*_ was applied to transition to the next state *s*_*t*+1_. As a result of the transition, reward *r*_*t*_ is received by the agent. *d*_*t*_ is a Boolean that indicates whether choosing *a*_*t*_ at *s*_*t*_ reaches the terminal state, a state which satisfies a predefined terminal condition. Reaching the terminal state marks the end of an episode where the state is reset to the initial state and the entire process repeats, to start a new episode. During the data collection, a training phase happens at a fixed frequency, between the transitions. During the training, a batch of transition data is randomly sampled and the q-network is updated according to the q-learning algorithm defined in Eq. (13).

While sharing a similar learning schematic as the previously mentioned three works, a different parameterization of free-form device structure was demonstrated in Ref. [196], using one-dimensional binary image space. Instead of setting action as changing the geometric measures, the authors defined action as adding or removing a structure in a binary gridcell representation that may change the topology of the structure, which is beyond the scope of mere geometric changes. Although the geometrical and topological approach may converge to the same point as the number of parameters increases, it is worth mentioning that topological exploration in a discrete space has led to a discovery of new properties such as the “high impact cell” described in Ref. [196].

Besides the value-based methods such as the q-learning, actor-critic framework [200] is also a popular formulation in reinforcement learning. In this framework, an actor and a critic are separate modules that work together to learn the value function and the policy as illustrated in the bottom panel of Figure 13A. As a type of one-dimensional freeform optimization, Wang et al. [201] applied the actor-critic framework and the q-learning to solve optimization of optical multi-layered thin films as sequence generation. Here, the authors used a variation of proximal policy optimization (PPO) [202] algorithm to design a thin multi-layered film that can be used to control absorption and reflection of lights of specific wavelengths. The optimized structures showed improved performances by achieving higher FoM and thinner and simpler layers compared to the baseline structures suggesting a promising feasibility of applying RL to sequence generation tasks in the optics field.

The successes demonstrated by the works in nanophotonics that adopted RL show the possibilities of applying further expanding RL in the field of nanophotonics. Additionally, different variations of DQN and other RL frameworks such as the actor-critic frameworks have shown improvement over the original DQN. These suggest that together with the expressivity of the deep neural networks, RL may be used to tackle problems in nanophotonics with higher complexity.

### 6.4 Physics-assisted approach

So far in this section we have covered the optimization methods using neural networks, whose training and inference are solely based on the data-driven methods. Despite many success stories, neural network-based optimization is inherently computation-heavy since it requires data from pre-calculated structure samples ranging from the order of 1000 to 100,000. The computational cost involved in a numerical electromagnetic simulation depends heavily on the size of the simulation space and also shows some dependence on the choice of simulation methods (most representatively, FEM, FDTD, and rigorous coupled-wave analysis [RCWA]). In general, a 2D electromagnetic simulation typically takes from a few seconds to minutes, but a 3D simulation often takes a few tens of minutes to even a few days due to the large mesh number. Costly data leads to incorporation of data augmentation processes which typically utilizes the translational or rotational symmetry of the device [203, 204].

Besides the data augmentation that was first developed in the field of image recognition, data efficiency of optimization problems in nanophotonics can be increased when considering the fact that the data being analyzed is based on a physical system. The simplest example of employing physical knowledge on neural networks can be found in a work by Tanriover et al. [205], where a physical intuition is used in designing a neural network structure. The physical structure consists of a cylinder placed periodically on a square lattice and the task was to predict and inverse design the intensity and phase of the transmitted beam. The authors used the fact that the optical response is unchanged if the dimensions of the cylinder scales with the wavelength of the impinging beam, which reduces the number of inputs of the system. They have confirmed that the performance of the inverse design is enhanced by utilizing such network configuration.

**Figure 14:**
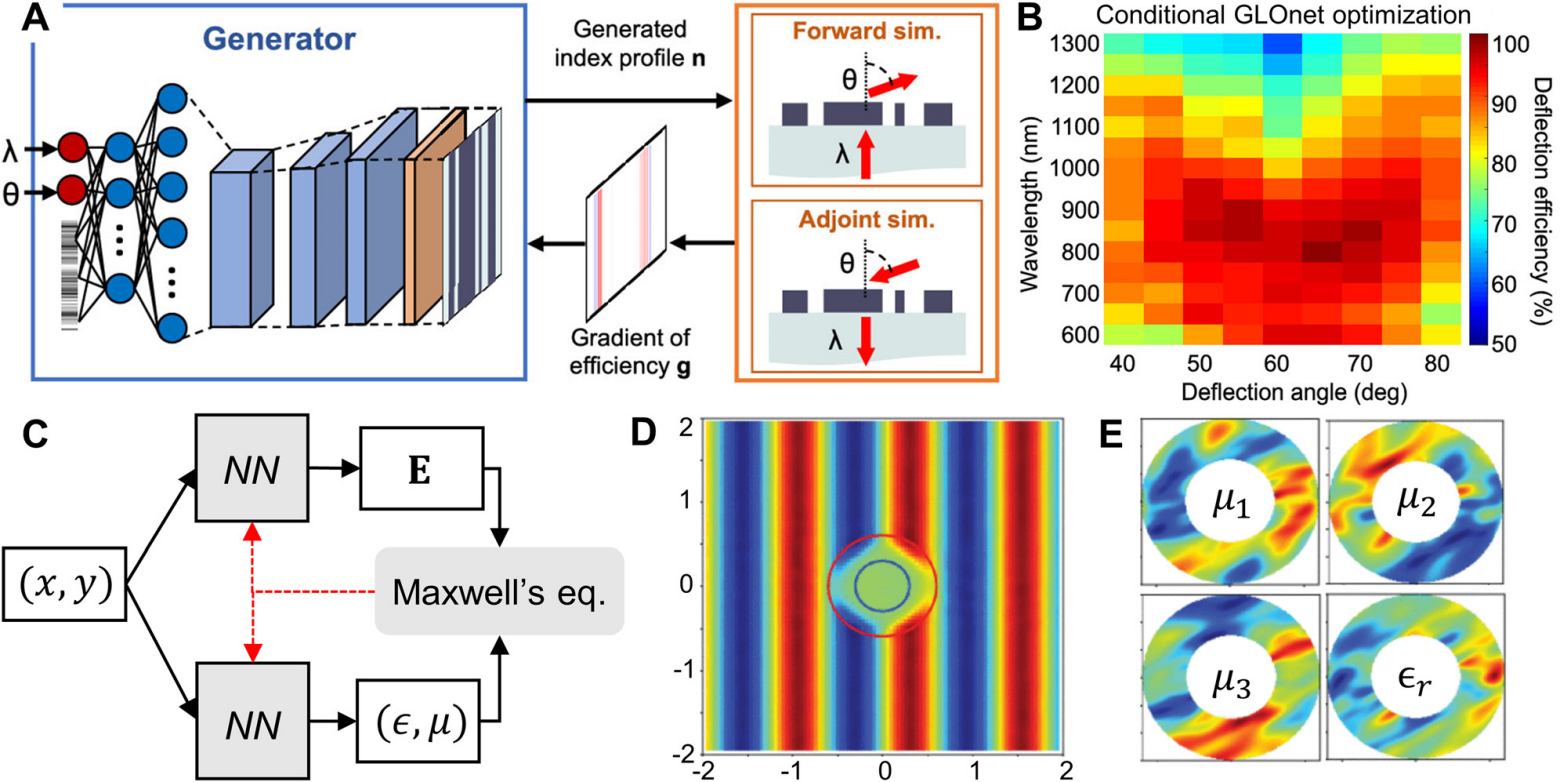
Use of physics-assisted neural network models in free-form nanophotonic device designs. (**A**) A generative neural network for designing a one-dimensional beam deflector with high efficiency. The generation network uses gradients from adjoint simulation for network training. (**B**) The optimized results from the generative neural network in (**A**). **A, B** adapted with permission from [128]. Copyright 2019, American Chemical Society. (**C**) A schematic depicting the neural network for the design of metamaterial (invisibility cloak). Top neural network maps the position coordinates to the field at the corresponding point while the bottom one maps to the permittivity/permeability of the metamaterial. (**D**) Spatial field distribution in the simulation space. Area between the red and blue circle represents the location where the invisibility cloak resides. (**E**) Permittivity and permeability distribution of the invisibility cloak. **D**, **E** adapted with permission from [217]. Licensed under CC BY 4.0.

The gradient of an objective function with respect to the design geometry can be obtained from the adjoint formulation. In Section 5, we focused on how this gradient can be directly applied to the design parameters to perform an adjoint-based optimization. However, this sometimes results in the device geometry failing to escape a local minimum and the optimization has to start from the very beginning (cold start) if a different initial geometry or different wavelength is used [28]. To bypass this issue, Jiang et al. [128] introduced a method to use adjoint simulation to train a generative neural network as shown in Figure 14A. They created a neural network that takes an operating wavelength, a deflection angle, and a random noise vector as inputs and generates the geometry of a high-efficiency 1D metagrating beam deflector. Weight of each neuron was adjusted by backpropagation using the gradient calculated with adjoint method, and more emphasis was put on high-efficiency devices by taking a weighted average proportional to the exponential of the efficiency. Through this approach, the neural network was able to learn the nonlinear relationship between the device geometry and optical response. Since a wavelength and a deflection angle were used as conditional inputs to the generator, the neural network was capable of creating the design of efficient metagrating corresponding to each desired wavelength and deflection angle. The efficiencies of the generated metagrating are comparable to the outcome of the adjoint-based optimizations within the range of physical parameters that the network was trained, as shown in Figure 14B.

A numerical solver for calculating the vector field distribution of a physical system utilizes the governing equation of the system. Schrodinger’s equation would be the governing equation for a quantum system, continuity/Navier–Stokes equations would be the governing equations for fluid dynamics, and it would be the Maxwell’s equations which govern the nanophotonic system. If a neural network is designed to obtain the vector field distribution of the entire system, the output fields should be in a good agreement with the governing equation. Raissi et al. [206, 207] pioneered this possibility in the field of quantum mechanics and fluid dynamics. They suggested a path to add a restriction to the network output as a form of loss term in the neural network as illustrated in Figure 10D. The additional loss term in such a physics-informed neural network (PINN) measures how much the output fields deviate from the governing equations. Application of PINN drew attention in many branches of science including fluid dynamics [208], [209], [210], [211], [212], plasma physics [213], material sciences [214], and even in geophysics [215, 216]. Nanophotonics was no exception.

Work by Fang et al. [217] is one of the first to use PINN toward a free-form inverse design problem in photonics. In the work, they first attempted to predict the electric field distribution in a vacuum medium by using the field distribution at the boundary as an only input. The boundary condition is the only information required to identify the field distribution inside the domain, according to the electromagnetism uniqueness theorem. The authors utilized a fully connected neural network that takes the position coordinates as an input and predicts the electric field intensity at that specific point as an output as illustrated in Figure 14C. In their work, the objective function for the training of the neural network was a simple sum of (1) a Maxwell residue which measures how much the output fields deviate from Maxwell’s equation and (2) mismatch between the given boundary condition and the prediction from the neural network. Although the Maxwell residue term requires the calculation of the partial derivatives of the electric field with respect to the position coordinates, the trained neural network predicted the electric field distribution with high accuracy. Additionally, an inverse problem of designing an invisibility cloak was tackled with a similar scheme. They created an additional neural network which predicts the permittivity and permeability distribution inside the cloaking metamaterial region. The values of permittivity and permeability were adjusted by the Maxwell residue loss term, thereby tuning the weights in the neural network. As a result, the optimized design showed a perfect cloaking performance as shown in Figure 14D. The corresponding permittivity, and permeability distributions for the optimized invisibility cloak are shown in Figure 14E. Although the inverse designed structure was practically impossible to fabricate due to non-existence of materials with corresponding permittivity/permeability, the inverse design scheme used in this work is versatile and can be adopted in a wide range of device design problems.

## 7 Considerations for fabrication errors

So far, we have discussed a variety of free-form nanophotonic design methods ranging from classical optimization methods to contemporary machine learning techniques. Although these methods produce optimized device designs that are supposed to exhibit high-performances in principle, it is often practically difficult to create the devices exactly identical to the optimized blueprints due to the imperfections in nanofabrication processes. In nanophotonics, small fabrication errors sometimes cause a catastrophic degradation of device performances, especially for the devices employing tiny “islands” or narrow gaps with highly concentrated electromagnetic fields [196]. Moreover, without proper restrictions on the device topology, a free-form optimization can also produce device designs that can never be fabricated even with modern lithography technologies. Therefore, considerations for fabrication errors are necessary to properly finalize the free-form optimization procedure. In this section, we discuss two representative approaches to achieve robustness against fabrication imperfections: density-filter-based method and level-set-based method.

### 7.1 Density-filter-based method

The density-filter-based method uses three levels of design representations, which consist of a design pattern, a filtered pattern, and a physical pattern. A design pattern *ρ* is a grayscale material distribution composed of continuous values ranging from 0 to 1, which are defined at every pixel of the design domain. Here, 0 means empty and 1 means filled state and thus the values in between do not have concrete physical meaning. A filtered pattern $\tilde{\rho }$ is obtained by applying a blur filter on the design pattern. The blur filter was introduced as a possible solution of “checkerboard” problems [122], in which the optimized structure has material and void elements weaved as a checkerboard. The application of filter to the design pattern *ρ* typically results in a blurred image of it, naturally preventing isolated features that are too small. Finally, a physical pattern $\bar{\rho }$ is obtained by binarizing the filtered pattern $\tilde{\rho }$ by applying a threshold filter [218, 219]. The threshold filter pushes a pixel to be void (0) if the input value was lower than a predefined threshold *η*, and fill up the pixel with a material (1) for the input values higher than *η*.

**Figure 15:**
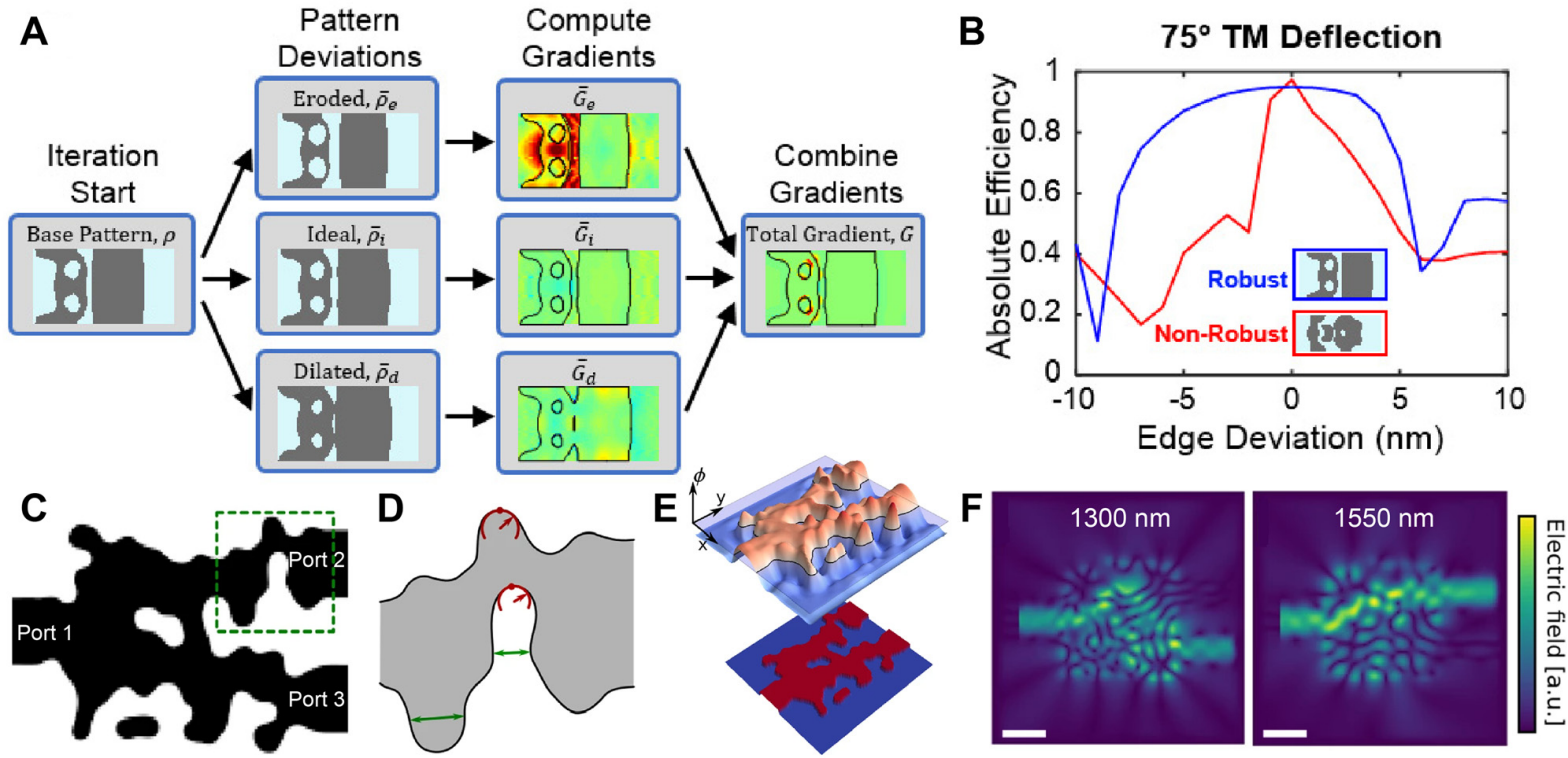
Methods for imposing fabrication constraints into free-form optimization and its results. (**A**) Three-level robustness-control scheme based on density filter, applied to beam deflector optimization. (**B**) Absolute efficiency dependence on edge deviation of optimized structures, with and without robustness-control. Inset shows final optimized device structures for both cases. **A**, **B** adapted with permission from [33]. Copyright 2019, Optical Society of America. (**C**) Schematic of binary represented waveguide demultiplexer. Port 1, 2, and 3 are input, desired output for 1300 and 1500 nm, respectively. (**D**) Magnified view of green box placed in (**C**). Red arcs and green lines show curvature and gap of the structure shown in (**C**), respectively. (**E**) Illustration of the level set function corresponding to the structure of (**C**) (top). The zero-level contour of the level set function decides the boundary of the physical structure (bottom). (**F**) Electric field intensity plot of the optimized structure at wavelengths of 1300 nm (left) and 1550 nm (right), respectively. The scale bar is 0.5 µm. **C**–**F** adapted with permission from [126]. Licensed under CC BY 4.0.

The robustness control using the density-filter-based method considers the fabrication errors in an etching process. As shown in Figure 15A, it assumes the etching process results in either three of the following cases: eroded, ideal, and dilated patterns. The ideal pattern refers to the pattern which uses a threshold value of *η* = 0.5. The eroded pattern refers to the pattern which is an over-etched version of a desired device pattern, having more void regions than it should be (*η* = *η*_0_ > 0.5). The dilated pattern, on the other hand, refers to the pattern which is an under-etched version of a desired device pattern, having more material regions than it should be (*η* = 1 − *η*_0_ < 0.5). The FoM gradients with respect to pattern variables are calculated for all three physical patterns and then the calculated gradients are combined and averaged to yield a total gradient that is finally used for updating the design pattern. The gradient for design field, *dF*/*dρ*, can be obtained from the gradient for physical field, $dF/d\bar{\rho }$ , by using chain rule, $dF/d\rho =\left(dF/d\bar{\rho }\right)\left(d\bar{\rho }/d\tilde{\rho }\right)\left(d\tilde{\rho }/d\rho \right)$ , where $d\bar{\rho }/d\tilde{\rho }$ and $d\tilde{\rho }/d\rho$ can be obtained by analytically differentiating the blur filter and the threshold filter functions. Through this approach, the FoM for the eroded, ideal, and dilated patterns will be simultaneously optimized, resulting in satisfactory performances for all cases. Wang et al. [33] applied this approach to design free-form metasurface beam deflectors that are robust against fabrication errors. As shown in Figure 15B, for the structure edge deviation of −5 to 5 nm, the design with robustness control shows over 80% absolute diffraction efficiency, while the performance of the design without robustness control rapidly decreases once the structure deviates from the ideal shape. We would like to recommend Ref [41, 125] to readers who seek for more detailed description on the density-filter-based robustness control method.

### 7.2 Level-set-based method

Another major approach to enforce robustness in the structure design is a level-set-based method. The special characteristic of the level-set method differentiated from other representation techniques is that it utilizes implicit functions to parameterize a device structure. This implicit parameterization allows easy and precise handling of topology changes applied to the boundaries of the structure. In the level-set method, the boundary of the structure is represented by the zero-level contour of level-set function (LSF) *ϕ*. The area having positive LSF value represents the material part of the structure, and the area having negative LSF value represents the void part. A LSF *ϕ* is a real-valued function, constructed by a sum of auxiliary basis functions. Commonly, locally polynomial spline functions or radial basis functions are used for the basis functions [47, 220]. By expressing an LSF as a linear combination of basis functions centered at different spatial locations, the zero-level contour of the LSF can possess topologically free-form outlines. Note that in the level-set method, the variables being optimized during optimization process are the LSF parameters *s*. The LSF parameters *s* can be the centers, scalings, radii, or other geometric parameters describing the basis functions. The change of these parameters results in evolution of zero-level contour of *ϕ* (material interface). During device optimization, the LSF *ϕ* is evolved by Hamilton-Jacobi equation [221, 222]: $\phi_{t}+v_{n}\left|\nabla \phi \right|=0$ , where *ϕ*_*t*_ represents time-derivative of LSF and $v_{n}=v\cdot n$ denotes the normal velocity field. The time *t* originally meant a physical time in its original form, but it has a slightly different meaning in the level-set scheme. Since the LSF is updated at each iteration, it is natural to conceive the time *t* as an iteration or step of the optimization. The normal vector **n** can be directly calculated as n=∇ϕ/|∇ϕ| , and the velocity field **v** can be obtained from shape sensitivity analysis [223], [224], [225], [226], [227]. Through the sensitivity analysis, the velocity field **v** is chosen such that the evolution of material interface conforms to the change of FoM, conceptually making it as a functional derivative of FoM upon the LSF *ϕ* [228]. The detailed derivation of shape sensitivity for electromagnetics is beyond the scope of this review. The shape sensitivity formulation using adjoint methods can be found in [43, 116, 127].

Vercruysse et al. [126] demonstrated a methodology of imposing analytic minimum feature size constraints integrated with the level-set method. In their work, a waveguide demultiplexer (WDM), whose structure is schematically shown in Figure 15C, is being optimized to transmit the light incident at Port 1 through Port 2 and Port 3, at 1300 and 1550 nm, respectively. This work uses analytic quantification of the curvature (red arcs in Figure 15D) and the gap size (green lines in Figure 15D) of the device structure as a function of LSF *ϕ* [229]. The LSF corresponding to the device structure shown in Figure 15C, is illustrated in Figure 15E. The major advantage of applying analytic minimum feature size constraints into the optimization problems is that the constraints can be added to the FoM as a form of penalty functions. Consequently, to minimize the penalty functions, the structure boundary spontaneously evolves toward the solution candidates obeying minimum feature size constraints. In terms of computational load, the level-set-based method is superior to the density-filter-based robustness-control method, considering that the latter inevitably needs to simulate three cases of eroded, ideal, and dilated pattern per iteration, whereas the former method does not change the number of simulations per iteration. For the optimization procedure, the authors started from grayscale material distribution, and performed continuous optimization. During the continuous optimization, the material distribution is binary-pushed using methods similar to the threshold filter described in the density filter scheme above. The binarized structure is used as an initial point of level-set method-based boundary optimization. The boundary optimization removed small features having smaller sizes than the minimum feature size constraint, resulting in fabrication-ready blueprint. As a result, the 2D optimized WDM structure showed transmission efficiencies of 93 and 92% at 1300 and 1550 nm in simulations, respectively, as it is also evident in the simulated electric field distributions shown in Figure 15F. The fabricated device designed by the same methodology showed 79 and 59% transmission efficiencies at 1300 and 1550 nm in experiments, respectively. It is worth noting that in their work, the performance comparison between the WDM structures optimized with different minimum feature sizes indicates that the smaller minimum feature size yields WDM designs with better performance. This observation coincides with our intuition that smaller minimum feature sizes unlock a broader range of high-performance, free-form solution space.

## 8 Outlook

It is clear that free-form optimization methods have already made a big impact on and will continue revolutionizing nanophotonic device design by enabling access to vast design spaces that have never been explored previously. Yet, unleashing its full potential would require further reducing the computational load by making the data generation (i.e. electromagnetic simulation) time faster and by increasing sample efficiency through intelligent search algorithms.

Optimization in a free-form design space often involves a massive computational load. Population-based optimization methods rely on trial-and-error over many candidates, which require a large computational budget. The adjoint-based method, in contrast, only requires forward and adjoint simulation to calculate adjoint sensitivity of the design space, but it often involves a number of “cold starts” with different choices of initial parameters to discover a global optimum. Machine learning techniques are a data-driven method requiring a larger dataset of simulated results. In these free-form design methods, calculating the full-wave solution to Maxwell’s equations could be a key bottleneck, hence it is desired to accelerate this calculation using some creative approaches. For example, a graphics processing unit (GPU) can speed up the calculations for solving Maxwell’s equations. As a GPU is specialized in parallel computing, some algebraic calculations of the full-wave electromagnetic simulation can be efficiently parallelized in a massive GPU environment. The acceleration of a FDTD simulation using GPU was examined in multiple papers [230], [231], [232], while similar attempts were made in FEM [233]. A boundary integral equation-based Maxwell solver, on the other hand, has been proposed as a way to considerably reduce the computing cost of the adjoint optimization approach [234]. When used in tandem with several of the optimization approaches outlined in our paper, it has the potential to greatly reduce direct simulation time, and therefore to speed up the entire optimization process.

As an alternative and auxiliary way of alleviating the computational load associated with rigorous electromagnetic simulations, one could consider employing neural networks to predict electromagnetic field distributions as *fast-yet-approximate* simulators. PINNs of Section 6.4 are the most attractive platform for this purpose. In comparison to the fields of fluid dynamics and plasma physics, where the use of a PINN as an alternative solver has been extensively studied, the use of a PINN for a photonic system is still in its infancy in nanophotonics. The computational advantages of PINN would become more evident for a three-dimensional simulation space since the conventional full-wave simulators are bound by the memory limitations. Furthermore, the intrinsic properties of Maxwell’s equations suggest that PINN can provide additional advantages in nanophotonics: for instance, a neural network can be jointly regulated by the physical residue and the adjoint simulation using the Lorentz reciprocity.

As a final remark, it should be noted that the sample efficiency of optimization algorithms should be further improved. There are two ways to use less data to enhance the sample efficiency: extracting information from previously obtained data, and focusing on collecting more useful, informed data. The former may be achieved by increasing the reusability of neural networks using transfer learning [235, 236] to gain knowledge from elsewhere. The latter may be achieved through reinforcement learning, which is less explored in the field of photonics and can be seen as an intelligent search algorithm. Many of the recent breakthroughs in the field of machine learning were based on the RL approaches – AlphaGo defeated a professional human Go player for the first time [152], AlphaFold has helped solve the protein folding problem [153], and chip placements were designed with RL [237]. In a similar manner, we expect to see breakthroughs originating from RL models that can solve large-scale nanophotonic device optimization problems, which were originally considered to be too complex to be handled with conventional methods.
